# NDP52 activates nuclear myosin VI to enhance RNA polymerase II transcription

**DOI:** 10.1038/s41467-017-02050-w

**Published:** 2017-11-30

**Authors:** Natalia Fili, Yukti Hari-Gupta, Ália dos Santos, Alexander Cook, Simon Poland, Simon M. Ameer-Beg, Maddy Parsons, Christopher P. Toseland

**Affiliations:** 1 School of Biosciences, University of Kent, Canterbury, CT2 7NJ UK; 2 Randall Division of Cell and Molecular Biophysics, King’s College London, Guys Campus, London, SE1 1UL UK

## Abstract

Myosin VI (MVI) has been found to be overexpressed in ovarian, breast and prostate cancers. Moreover, it has been shown to play a role in regulating cell proliferation and migration, and to interact with RNA Polymerase II (RNAPII). Here, we find that backfolding of MVI regulates its ability to bind DNA and that a putative transcription co-activator NDP52 relieves the auto-inhibition of MVI to enable DNA binding. Additionally, we show that the MVI–NDP52 complex binds RNAPII, which is critical for transcription, and that depletion of NDP52 or MVI reduces steady-state mRNA levels. Lastly, we demonstrate that MVI directly interacts with nuclear receptors to drive expression of target genes, thereby suggesting a link to cell proliferation and migration. Overall, we suggest MVI may function as an auxiliary motor to drive transcription.

## Abstract (web summary)

Myosin VI (MVI) is known to interact with RNA Polymerase II and to play non-cytoplasmic roles in cells. Here, the authors provide evidence that the transcription co-activator NDP52 regulates MVI binding to DNA and that MVI interacts with nuclear receptors to drive gene expression.

## Introduction

Myosins are molecular motors that perform vital roles in a plethora of cellular processes. Myosin VI (MVI) is a unique member of the myosin family with the ability to move towards the minus end of actin filaments^1^. This property enables MVI to be involved in cell migration, endocytosis, exocytosis and transcription^2, 3^. The functional diversity of MVI relies on its tightly regulated association with various binding partners. Given its multi-potent nature, malfunction of MVI leads to various diseases including cardiomyopathy, deafness and cancer^4–7^.

**Fig. 1:**
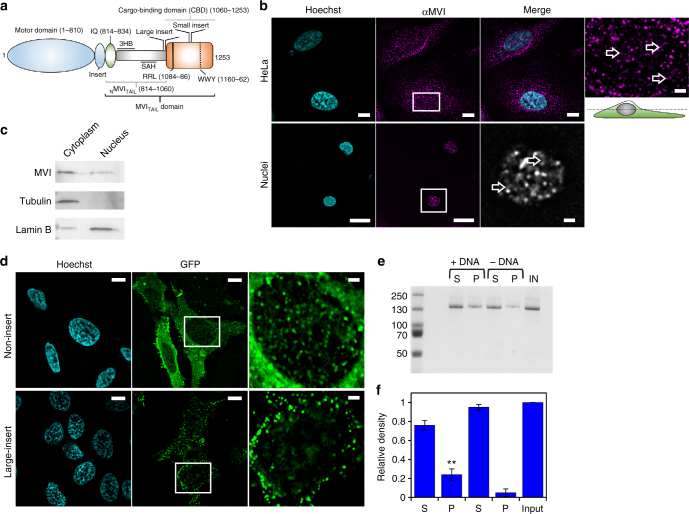
Myosin VI is distributed throughout the nucleus. **a** Cartoon depiction of the MVI domains and key features discussed in the text. **b** Immunofluorescence staining against MVI (magenta) and DNA (cyan) in HeLa cells and isolated nuclei (see Supplementary Fig. 1 for nuclei images). Arrows highlight filamentous structures within the nucleus. Images were acquired at the mid-point of the nucleus. Scale bar 10 μm for whole images and 1 μm for inserts. **c** Western blot against MVI following HeLa cell fractionation. Tubulin and lamin B are used as cytoplasmic and nuclear markers, respectively. **d** Representative images of transiently expressed NI- and LI-GFP-MVI in HeLa cells combined with DNA staining (cyan) (Supplementary Fig. 3). Images acquired as in B. **e** Pull-down of recombinant MVI using a 500 bp DNA substrate. **f** Plot of band intensities normalised to the input sample (5 μM). Errors bars represent SEM from three independent preparations. (**p < 0.001 by two-tailed *t*-test between presence and absence of DNA)

MVI comprises a motor domain, followed by a neck region consisting of a unique insert, which confers the reverse directionality, and an IQ domain (Fig. 1a). The N-terminal tail domain (_N_MVI_TAIL_) contains two structural domains a three-helix-bundle (amino acids 835–916)^8^ (3HB) and a single-alpha-helix (amino acids 942–978) (SAH)^9^. The C-terminal tail domain contains the globular cargo binding domain (CBD). In addition, two regions within the tail can be alternatively spliced resulting in a 31-residue insertion (large insert, LI) before the CBD, and/or an 8-residue insertion in the CBD (small insert, SI). This leads to four splice isoforms, the non-insert (NI), SI, LI and LI + SI^10^.

The CBD confers specificity for cargo through its ability to bind partners, such as Dab2, GIPC and NDP52, at two motifs WWY and RRL^11–13^. NDP52 was initially identified in the nucleus^14, 15^ but has been better characterised in cell adhesion and autophagy^11, 16^. Intriguingly, NDP52 shares 70% sequence identity to its family member CoCoA, a known transcription co-activator, which suggests a potential role in transcription^17^.

MVI is overexpressed in aggressive cancers including ovarian, breast and prostate^5, 7, 18^, and depletion of MVI leads to decreased cell motility and/or proliferation^18–20^. As MVI is known to have roles in gene expression^3, 21^, it is possible that this is linked to the nuclear role of MVI.

To shed light upon this hypothesis, here we have explored the potential functions of nuclear MVI. The association of MVI with RNAPII is dependent upon its DNA binding, which is in turn regulated by co-activator NDP52. This alters the MVI oligomeric and mechanical properties. Moreover, MVI interacts with nuclear receptors for specific gene targeting. We suggest MVI is an auxiliary motor for RNAPII to drive gene expression.

## Results

### Myosin VI non-insert isoform is recruited to the nucleus

Before exploring the nuclear role of MVI, we first assessed its distribution. Immunofluorescence on both HeLa cells, and isolated nuclei, and cell fractionation confirmed an endogenous nuclear population (Fig. 1b, c and Supplementary Movies 1–5), consistent with previous reports^3^. MVI was distributed throughout the nucleus, although occluded from the nucleoli. MVI decorated punctate and filamentous structures, which occasionally interconnected forming small networks (Fig. 1b, Supplementary Movies 1 and 2). To explore the nature of these structures, we tested the possibility of MVI localising on nuclear actin filaments. Dense nuclear actin cables were absent in whole cells (Supplementary Fig. 2a and Supplementary Movie 6), but a sparse actin network may be present. However, the lack of staining in the isolated nuclei (Supplementary Fig. 2a) suggested that MVI structures are not actin-based but may represent MVI bound to chromatin.

Recently, it was shown that several cancers only express the NI isoform^22^, as is the case in HeLa cells. Yet, it has not been shown if the isoform selection and nuclear localisation are functionally linked. To address whether such a relationship exists, we compared the intracellular distribution of transiently expressed EGFP-NI- and LI-MVI. EGFP-NI-MVI had a similar distribution to the endogenous protein (Fig. 1d). Strikingly, EGFP-LI-MVI localisation was restricted to the cell periphery (Fig. 1d and Supplementary Fig. 3a, b).

The LI has been recently shown to encode an alpha-helix that blocks the RRL-motif, establishing an isoform-specific regulation for the interactions with binding partners^22^. A point-mutation within the LI, namely M1062Q, disrupts this process. As expected, EGFP-LI-MVI(M1062Q) had a similar cellular distribution to the NI isoform (Supplementary Fig. 3c). Taken together, these data demonstrate that splicing regulates the recruitment of MVI into the nucleus, likely by controlling access to the RRL-motif.

### Myosin VI binds DNA through its CBD

**Fig. 2:**
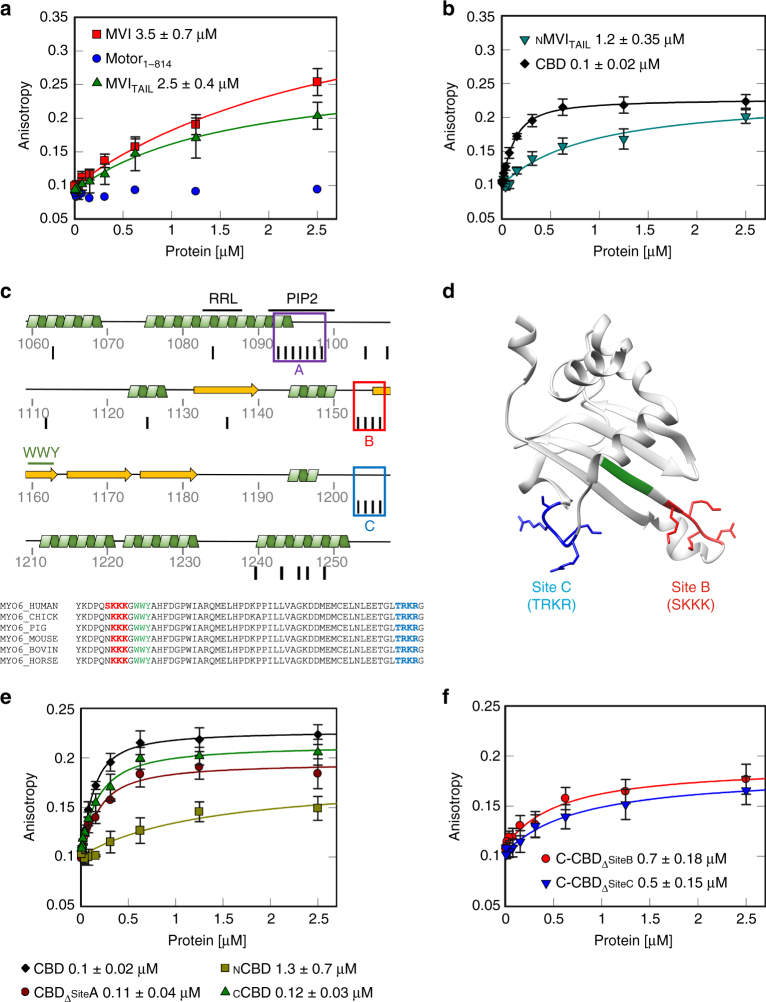
DNA binding by myosin VI. **a**, **b** Fluorescence anisotropy titrations of MVI domains against a 40 bp fluorescein amidite (FAM)-DNA (50 nM). Data fitting was performed as described in Methods (*K*_d_ ± SEM *n* = 3 independent experiments). Due to the low binding affinity, Motor_1-814_ could not be fitted using the model. **c** Cartoon depicting the secondary structure within the CBD. Binding partner motifs and known lipid binding sites are highlighted along with three predicted clusters of DNA binding. Black lines represent residues predicted to be involved in DNA binding. Sequence alignment shows the conservation of sites B and C. **d** Structure of _C_CBD with site B (red), C (blue) and WWY motif (green) (PDB:2KIA^54^). **e**, **f** Fluorescence anisotropy titrations of CBD constructs, as performed in **a**

The presence of MVI throughout the nucleus and its known association with RNAPII leads to the intriguing possibility that this myosin might bind to chromatin. BindN^23^ predicted sites for interaction with DNA are highlighted throughout MVI, with higher frequency within its tail (Supplementary Table 1 and Fig. 2b). To establish if MVI can interact with DNA, we used a DNA pull-down assay. Recombinant NI-MVI was isolated in complex with the linear DNA (Fig. 1e).

To further explore this interaction in a quantitative manner, we utilised fluorescence anisotropy. MVI was titrated against DNA revealing a relatively weak binding (Fig. 2a and Supplementary Table 2). To identify the regions within MVI that mediate DNA binding, the titrations were also performed using isolated domains. Our results demonstrate that the DNA-binding ability is restricted to the MVI_TAIL_ (Fig. 2a). Intriguingly, the isolated CBD displayed a 25-fold higher affinity for DNA (Fig. 2b). The CBD bound promiscuously to single-stranded and double-stranded DNA of varying composition, although weaker for substrates <20 bases (Supplementary Fig. 2c). The N-terminal MVI_TAIL_ (_N_MVI_TAIL_814-1060) also bound DNA, but not as strongly as the CBD (Fig. 2b). A summary of the kinetic parameters is presented in Supplementary Table 2.

To understand the DNA-binding mechanism, we used pre-steady-state measurements. Here, the CBD had a four-fold higher association rate constant than full-length (FL) protein, while the dissociation rate constant was essentially unchanged (Supplementary Fig. 2d and Supplementary Table 3). This indicated that once binding is achieved, the stability of the bound state is the same in both constructs. Therefore, we conclude that MVI binds DNA through its CBD and this binding is impeded in the FL-MVI, possibly due to the adopted conformation.

### DNA binding occurs through two adjacent loops in the CBD

We decided to identify the sites conferring the DNA-binding ability. Based on predictions, we distinguished three clusters—sites A–C (Fig. 2c) in the MVI_TAIL_. We generated various MVI_TAIL_ mutants and truncations, and assessed their DNA-binding potential. Mutagenesis of site A, which corresponds to a known PIP_2_ lipid-binding site^13^, perturbed lipid binding (Supplementary Fig. 2e), but only partially affected DNA interactions (Fig. 2e). This suggested that binding occurs through sites B and C. This was further reinforced when we tested two truncations of CBD, _N_CBD_1060-1120_ (containing Site A) and _C_CBD_1121-1253_ (containing sites B and C) (Fig. 2e). _N_CBD displayed a considerably reduced DNA binding, while the _C_CBD was indistinguishable from the entire CBD domain. Sites B and C are located on two highly conserved adjacent loops which likely form a single binding surface in close proximity to the WWY motif (Fig. 2d). Alanine mutagenesis of either site reduced DNA binding (Fig. 2f), indicating that both sites are required for efficient interaction with the DNA. Mutagenesis of both sites destabilised the domain thereby preventing the measurements.

### Backfolding of myosin VI regulates its ability to bind DNA

**Fig. 3:**
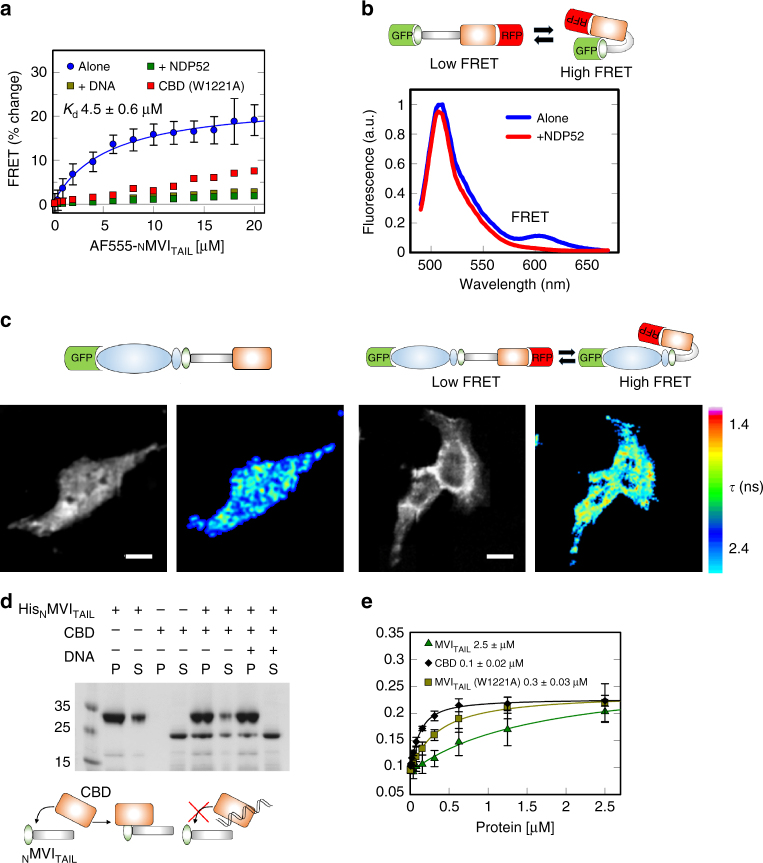
Regulation of myosin VI backfolding. **a** FRET titration of _N_MVI_TAIL_ against CBD (1 μM) or CBD(W1221A) in the presence of DNA, or NDP52, at 10 μM. Data fitting was performed as described in Methods. NDP52 and CBD(W1221A) data are plotted as disconnected points due to low signal change. See Supplementary Fig. 4a–c for raw intensity data. **b** Representative fluorescence spectra of 2 μM GFP-MVI_TAIL_-RFP ± 15 μM NDP52. **c** FLIM measurements of in vivo MVI conformational dynamics. Above, schematic showing the constructs. Left, GFP intensity and lifetime images. Right, intensity and lifetime images of the biosensor. Lifetime (*τ*) is represented by a pseudocolor scale. Scale bar is 20 μm. **d** Pull-down of CBD by His-tagged _N_MVI_TAIL_ both at 10 μM ± 20 μM 40 bp DNA. P and S represent pellet and supernatant fractions. **e** Fluorescence anisotropy titrations of MVI domains against a 40 bp FAM DNA, as performed in Fig. 2a

As shown above, although the CBD binds DNA efficiently, the binding ability of the FL protein is impeded. This suggests that DNA binding is regulated. MVI is suggested to regulate its activity through backfolding^24–26^. We therefore explored if such a mechanism regulates DNA association. Having already established that the MVI_TAIL_ displayed similar DNA binding behaviour as FL-MVI (Fig. 2a), we hypothesised that, if backfolding occurs, the tail should fold upon itself. Hence, we assessed whether the CBD and the _N_MVI_TAIL_ can form an interaction. To this end, a FRET-based assay was employed by titrating Alexa555-_N_MVI_TAIL_ to FITC-CBD. A significant change in FRET was detected, indicating that the two domains are in close proximity (Fig. 3a, dissociation constants are summarised in Supplementary Table 4). A fluorescence anisotropy assay confirmed this interaction (Supplementary Fig. 4d). To further confirm the ability of the MVI_TAIL_ to backfold, we used a dual-labelled construct, GFP-MVI_TAIL_-RFP. Here, FRET would report upon the folded state. Indeed, a small FRET signal was observed (Fig. 3b), indicating a degree of backfolding.

While we have explored how backfolding occurs in vitro, it is unknown if this occurs in vivo. To explore the in vivo conformation dynamics, we used fluorescence lifetime imaging (Fig. 3c). A GFP–MVI–RFP construct was used as a conformation reporter in HeLa cells. Indeed, two populations were observed in a high and low FRET state, indicating that backfolding does occur in vivo.

The _N_MVI_TAIL_–CBD complex represents the folded form, where we propose the DNA-binding sites are occluded. To test whether backfolding and DNA binding are mutually exclusive, we performed the in vitro FRET measurements with a pre-formed CBD–DNA complex. There was a lack of interaction between the _N_MVI_TAIL_ and CBD–DNA complex, suggesting that the presence of DNA prevented backfolding (Fig. 3a). To further confirm our results, all the above interactions were also assessed using pull-down assays (Fig. 3d). The CBD co-purified with _N_MVI_TAIL_, whereas the presence of DNA sequestered the CBD. Taken together, these data demonstrate that DNA binding is regulated through intra-molecular backfolding.

### Calcium does not regulate myosin VI backfolding

A mechanism controlling the open/closed state of the tail could be regulated by cofactors such as ATP, actin and Ca^2+^. However, DNA binding by MVI was not enhanced in their presence (Supplementary Fig. 4f), even though Ca^2+^-Calmodulin was recently reported to regulate the backfolding of MVI^24^. Pull-down assays in the presence of CBD, with and without calcium were performed using two truncations Motor_1-814_ and Motor_1-1060,_ containing 1 or 2 calmodulin binding sites, respectively. All constructs containing the motor domain were co-expressed with calmodulin. Supplementary Fig. 4g shows that both constructs associated with the CBD. Whilst expected for the Motor_1-1060_, binding to Motor_1-814_ suggested the CBD also forms extensive contacts with the motor domain, which is consistent with SAXS data by Spink^26^. However, Ca^2+^ had no discernible impact upon these interactions, in contrast  to Batters^24^. This discrepancy could be attributed to the fact that, in their work, the pull-down experiments, which were assessing the ability of the tail to backfold, were only performed in the absence of Ca^2+^. Instead, only far-dot western blots were performed in the presence of Ca^2+^. However, those experiments were addressing the effect of Ca^2+^ on the interaction of the tail with calmodulin, rather than the backfolding of the tail itself.

### Interaction with NDP52 regulates myosin VI backfolding

Having established that ATP, actin and Ca^2+^ do not regulate MVI backfolding, we explored the possibility that a binding partner could be an alternative regulator. The adaptor binding site, the RRL-motif, is situated away from sites B to C and, therefore, it is unlikely to perturb DNA binding. One such protein, which binds this motif, is NDP52. To address whether NDP52 is an MVI regulator, the FRET assay between the _N_MVI_TAIL_ and the CBD was repeated following pre-incubation of the CBD with NDP52. Similar to DNA, NDP52 sequestered the CBD preventing the interaction between the two domains (Fig. 3a). Moreover, addition of excess of NDP52 in our GFP–MVI_TAIL_–RFP reporter resulted in the loss of the FRET signal (Fig. 3b), indicating the tail’s unfolding.

For NDP52 to elicit unfolding of MVI, it would need to disrupt sites within the protein that form a stable interaction, such as the recently proposed W1221^27^. Mutagenesis lead to an increase in actin binding, which was attributed to unfolding of MVI. Using the FRET assay, we observed that CBD(W1221A) failed to bind to _N_MVI_TAIL_ (Fig. 3a), confirming that the mutation likely leads to the unfolding of the protein. Interestingly, the MVI_TAIL_(W1221A) was able to bind DNA, similar to the CBD (Fig. 3e), indicating that the adopted conformation enhances the ability of MVI to bind DNA. Taken together, our data suggest a model, whereby NDP52 triggers a conformational change of MVI leading to an unfolded state with enhanced DNA binding ability.

### NDP52 is a nuclear binding partner of myosin VI

While calcium and ATP are important regulators that fine-tune motor function, we show that NDP52 can trigger unfolding of MVI by relieving the autoinhibition between the CBD and the N-terminal tail/motor domain. In order to unravel the physiological relevance of our in vitro findings and explore the role of NDP52, we decided to characterise NDP52 and its interaction with MVI.

**Fig. 4:**
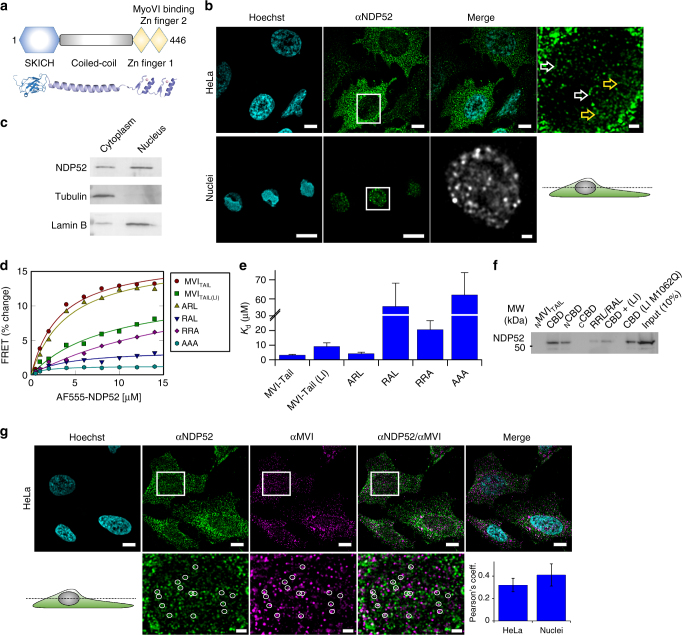
NDP52 is a nuclear myosin VI binding partner. **a** Cartoon and structural model depicting NDP52 domains as mentioned in the text. **b** Immunofluorescence staining against NDP52 (green) and DNA (cyan) in HeLa cells and isolated nuclei, as performed in Fig. 1b. Arrows highlight filamentous structures. Scale bar 10 μm for whole images and 1 μm for inserts. **c** Western blot against NDP52 following HeLa cell fractionation. Tubulin and lamin B were used as cytoplasmic and nuclear markers, respectively. **d** FRET titration of MVI_TAIL_ constructs against NDP52 (1 μM). Data fitting was performed as described in Methods giving a *K*_d_ as plotted in **e** (error bars represent SEM from three independent experiments). **f** Western blot against NDP52 following isolation from nuclear extracts by MVI_TAIL_ constructs. Loading controls for the recombinant proteins is shown in Supplementary Fig. 9. **g** Immunofluorescence staining against NDP52 (green), MVI (magenta) and DNA (cyan), as performed in Fig. 1b. Insert depicts co-localising foci. Scale bar 10 μm for whole images and 1 μm for inserts. Pearson’s coefficient is shown for HeLa and nuclei (Supplementary Fig. 5b). Error bars represent SEM from 10 images

NDP52 consists of a SKICH domain followed by a long coiled-coil and two Zinc finger domains that may confer the ability to bind DNA (Fig. 4a). Indeed, we observed that NDP52 can bind DNA (Supplementary Fig. 5a) with high affinity. This binding precluded the use of this protein in our DNA-binding assays because the contribution by MVI would be masked by that of NDP52. However, together with MVI’s ability to bind DNA and its nuclear distribution, this finding pointed towards the direction of a potential partnership between NDP52 and MVI in the nucleus.

We observed the distribution of NDP52 in HeLa cells using immunofluorescence and cell fractionation. Endogenous NDP52 was distributed throughout the cytoplasm as well as into the nucleus, similar to MVI (Fig. 4b, c). The nuclear pool of NDP52 decorated punctate structures, which were, in places, interconnected into small ring-like networks (Supplementary Movie 7). Similar distribution was also observed in isolated HeLa nuclei, suggesting that these structures are stable (Fig. 4b and Supplementary Movie 8). As with MVI, this may represent binding to chromatin.

To characterise the interaction between MVI and NDP52, we initially performed an in vitro FRET assay using FITC-MVI_TAIL_ and Alexa555-NDP52. NDP52 was titrated against MVI_TAIL_ containing various mutants of the RRL-site, as well as a LI-MVI_TAIL_ (Fig. 4d, e and Supplementary Table 4). Wild-type MVI_TAIL_ had a low micromolar affinity for NDP52. When we mutated the first arginine (ARL), there was essentially no effect upon NDP52 binding. Conversely, mutation of the second arginine (RAL) abolished the interaction, whereas removal of the leucine (RRA) led to a 10-fold decrease in affinity. Finally, the presence of the LI reduced binding by 4-fold.

This was complemented by pull-down assays using recombinant fragments of CBD as bait to search nuclear extracts for interactions with native nuclear NDP52 (Fig. 4f). As expected, binding occurred within the RRL-containing _N_CBD. In addition, whereas the presence of the LI compromised the binding to NDP52, this effect was rescued by the M1062Q mutant. Overall, these data suggest that MVI can associate with the nuclear pool of NDP52. Indeed, when we immuno-stained HeLa cells (Fig. 4g) against the endogenous MVI and NDP52, we observed that the two proteins partially colocalised within the nucleus: they occasionally localised on the same punctate and filamentous structures, sometimes as part of the same local networks. This partial co-localisation is consistent with the micromolar dissociation constant, and is indicative of a dynamic interaction. The co-localisation was also dependent upon the MVI isoform whereby the LI displayed the lowest colocalisation (Supplementary Fig. 5c, d). Therefore, taking all data together, we conclude that NDP52 is a nuclear partner of MVI.

### Interaction with NDP52 mediates myosin VI dimerisation

We have revealed how NDP52 and MVI interact in the nucleus to bring about unfolding of MVI, enabling DNA binding. However, this may not be the only consequence of this interaction. The oligomeric state of MVI has long been a controversial subject, along with the possible roles of partners^25, 28^. MVI contains a potential dimerisation site through a predicted coiled-coil^29^, just before the CBD. It is probable that this motif is also masked in the folded conformation, in a similar manner to the DNA-binding site. Therefore, the NDP52-induced unfolding of MVI could enable dimerisation, forming a highly processive motor.

To explore this hypothesis, we firstly assessed the ability of MVI to dimerise, using an actin pull-down assay. Here, MVI pellets when bound to actin, causing co-sedimentation of proteins that MVI is in complex with. Therefore, if the MVI_TAIL_ is incubated with FL-MVI and a heterodimer is formed, the MVI_TAIL_ will pellet with the MVI-actin complex. Indeed, MVI_TAIL_ was observed in the pellet (Supplementary Fig. 6a, b). The bound molar concentration of MVI_TAIL_ was limited by the sub-stoichiometry concentration of MVI. When performed with higher MVI concentrations, a greater amount of MVI_TAIL_ co-sedimented. Overall these data show that MVI can dimerise, albeit inefficiently.

**Fig. 5:**
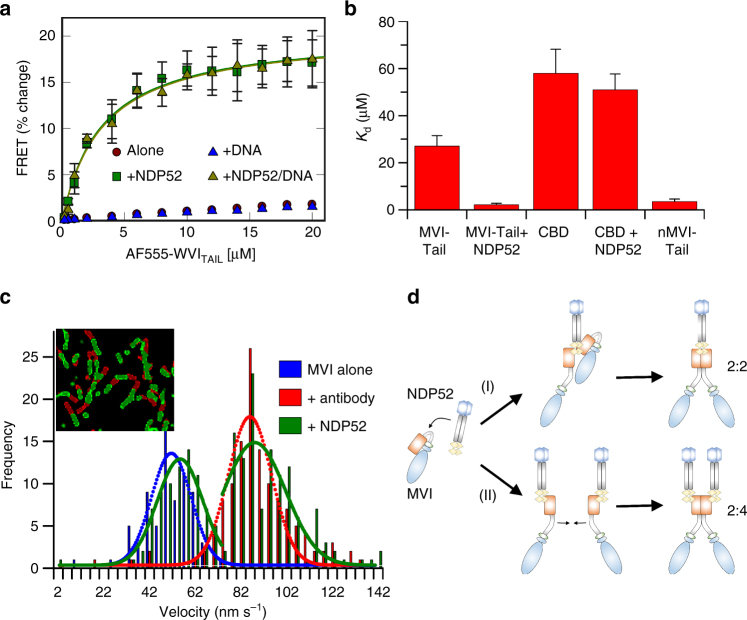
NDP52-dependent dimerisation of myosin VI. **a** FRET titration of FITC-MVI_TAIL_ against 1 μM AF555-MVI_TAIL_ ± DNA (20 μM) and NDP52 (20 μM). Data fitting was performed as described in Methods giving a *K*_d_ as plotted in **b**. See Supplementary Fig. 7a–c for raw intensity data. **b** Plot of *K*_d_ from titrations in **a** and Supplementary Fig. 7d–g (error bars represent SEM from three independent experiments). **c** Velocity histogram from sliding filament assay with MVI immobilised alone (blue), through antibody (red) and NDP52 (green). Insert shows first frame (red) and after 60 s (green). **d** Two routes of NDP52-dependent dimerisation with different stoichiometry. (i) NDP52 unfolds MVI then directly recruits a second molecule. (ii) Each MVI is unfolded by an individual NDP52

To quantify this process, we performed a FRET assay, whereby two pools of MVI_TAIL_ were labelled, one with FITC and the other with Alexa555. Titrations revealed a weak association (Fig. 5a, b and Supplementary Fig. 7d), consistent with our pull-down results. Interestingly, upon addition of excess NDP52, a large change in FRET was observed, indicating the formation of a stable dimer. Moreover, while the presence of DNA could not yield dimerisation, it did not prevent it from occurring in the presence of NDP52. This observation highlights an important distinction between DNA and NDP52; while both can sequester the CBD, only NDP52 can disrupt the interaction with the rest of the tail and promote unfolding.

### Dimerisation is an intrinsic property of the myosin VI

To determine whether dimerisation is driven by the coiled-coil domain located in the _N_MVI_TAIL_, the FRET assay was repeated using _N_MVI_TAIL_ and the CBD. A significant FRET change was observed for _N_MVI_TAIL,_ which was independent of its ability to bind NDP52 (Fig. 5b and Supplementary Fig. 7f). This confirmed the innate ability of MVI to dimerise within this region. The CBD did not dimerise, irrespective of NDP52 (Fig. 5b and Supplementary Fig. 7e, g). NDP52, and many other partners, are likely dimers themselves, and therefore, it has long been thought that MVI dimerisation is driven through its partner, rather than being innate to itself. When sub-stoichiometric amounts of NDP52 were used, upon saturation of NDP52 with CBD, a FRET change was observed (Supplementary Fig. 7g). This is likely to be due to the binding of two CBD monomers to each subunit of NDP52. This indirect dimerisation was not observed with the MVI_TAIL_ because NDP52 was in excess (Fig. 5a). However, when sub-stoichiometry NDP52 was used, a breakpoint in the titration was observed at a concentration equal to that of NDP52 (Supplementary Fig. 7h). This was followed by a linear increase in FRET, which is indicative of further binding, but in a different manner to the one observed in excess of NDP52. Therefore, our data suggest that both dimerisation mechanisms are possible (Fig. 5d), opening the intriguing possibility that localised partner concentration could regulate different complex formats, each with a potentially different role.

### Partner induced dimerisation regulates myosin VI motility

Dimerisation can alter the mechanical properties of the protein, which translates into changes in the motor behaviour. The steady-state ATPase kinetics of MVI revealed a 50% reduction in the rate constant following addition of NDP52 (Supplementary Fig. 6c), but no change in the presence of DNA. This is typical of molecular gating, whereby each head of the dimer alternates ATP hydrolysis^30^. Moreover, the rate constant for FL-MVI in the presence of NDP52 was essentially identical to that of the MVI truncation without CBD (Motor_1-1060_). We propose this construct would fail to backfold but could dimerise through the coiled-coil.

To explore the mechanical consequences of MVI dimerisation, sliding filament motility assays were performed (Fig. 5c). Antibodies to the MVI_TAIL_ are used to immobilise MVI in the correct orientation, in order to enable translocation of actin filaments. This approach resulted in an increased velocity from 40–50 to 90 nm s^−1^. However, antibodies were not required if MVI was pre-incubated with NDP52. Here, two populations were observed with velocities at 50–60 and 90 nm s^−1^, which was dependent upon the NDP52 concentration (Supplementary Fig. 6d). We suggest this corresponds to apo- and NDP52-bound MVI, respectively. As we have shown, MVI exists in a folded conformation, where it would be unlikely to generate efficient motility, as revealed by the low velocity population. However, NDP52 unfolds MVI, inducing the mechanical activation of the motor.

Taking all our data together, we propose the following model (Fig. 5d): NDP52 initially associates with MVI in its folded state. This triggers unfolding of the protein and subsequent recruitment of a second myosin. Dimerisation could occur through two mechanisms: dimerisation of two MVI monomers around a single partner dimer, or dimerisation of two myosin molecules, each bound to a different partner molecule.

### The nuclear role of NDP52 as a transcription regulator

**Fig. 6:**
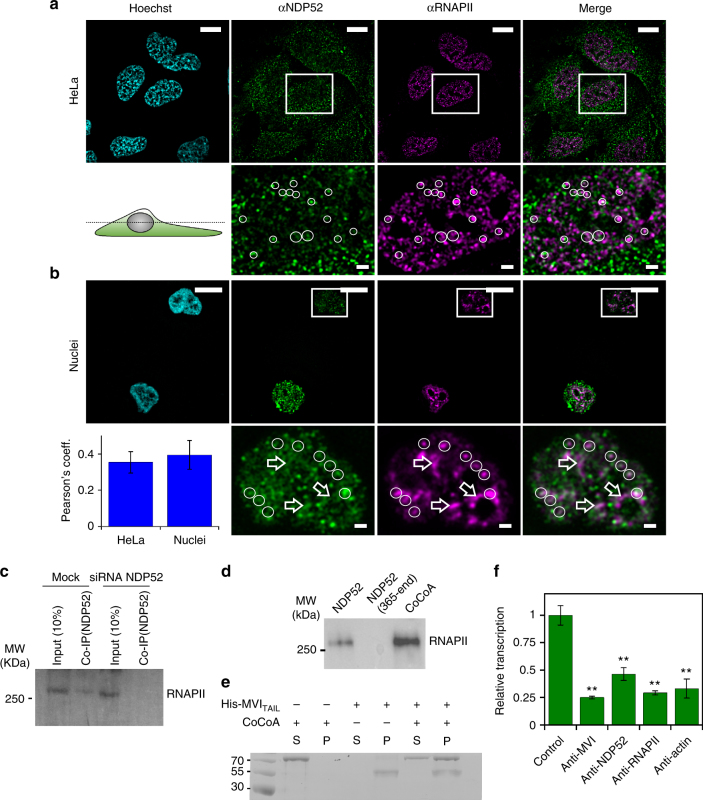
NDP52 transcription regulation. **a** Immunofluorescence staining against NDP52 (green), RNAPII (magenta) and DNA (cyan), as performed in Fig. 1b. Insert depicts colocalising foci. Scale bar 10 μm for whole images and 1 μm for inserts. **b** Immunofluorescence on HeLa nuclei as in (a)  with Pearson’s coefficient (error bars represent SEM from 10 images). **c** Co-immunoprecipitation of NDP52 and RNAPII, using an antibody against NDP52 to immunoprecipitate RNAPII from HeLa cells. Immunoprecipitation does not occur in HeLa cells subjected to siRNA knockdown of NDP52. **d** Western blot against RNAPII following isolation from nuclear extracts by NDP52 constructs and CoCoA. Loading controls for the recombinant proteins is shown in Supplementary Fig. 9. **e** Pull-down of CoCoA by His-tagged _N_MVI_TAIL_ both at 5 μM. P and S represent pellet and supernatant fractions. **f** In vitro transcription by HelaScribe extracts following antibody depletion as described in Methods. Samples were normalised to a non-depleted control reaction (error bars represent SEM **p < 0.001 by two-tailed *t*-test) from 5 independent experiments (Supplementary Fig. 8a, b)

Based upon its similarity to transcription co-activator CoCoA and its binding to MVI, we explored if NDP52 partners MVI in transcription. The distribution of the NDP52 in relation to RNAPII was examined using immunofluorescence (Fig. 6a, b). NDP52 partially colocalised with RNAPII on punctate structures. Moreover, using recombinant NDP52 as bait in nuclear extracts, we confirmed that FL-NDP52 formed a complex with RNAPII. This was further supported by co-immunoprecipitation of RNAPII by NDP52 from HeLa extracts (Fig. 6c, d).

The MVI-binding site is conserved between NDP52 and CoCoA, and a pull-down between recombinant proteins revealed the two form a stable interaction (Fig. 6e).

To further explore the role of NDP52 in transcription, we performed in vitro transcription assays using the HeLaScribe extracts. To assess the role of various proteins in transcription, we used antibody depletion to sequester selected proteins from the extracts, before performing the transcription assays (Fig. 6f and Supplementary Fig. 8a, b). As expected, depleting a pool of RNAPII led to a decrease in transcription. Moreover, depletion of actin which is known to be bound to the RNAPII complex^31, 32^, also decreased transcription. Consistent with previous findings under the same conditions^3^, transcription was decreased to about 25% upon depletion of MVI. Interestingly, a 50% decrease was observed when NDP52 was depleted, suggesting that this protein is indeed involved in transcription.

### Myosin VI’s interaction with RNA polymerase II

MVI has previously been associated with sites of active transcription^3^. Here, we have illustrated that MVI regulator NDP52 also colocalised with RNAPII and enhanced transcription.

**Fig. 7:**
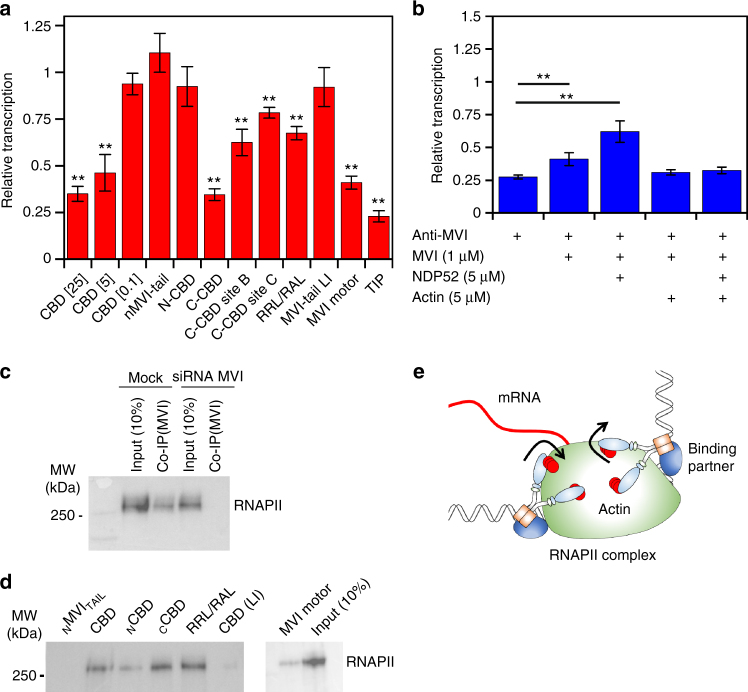
Coupling myosin VI to the RNAPII complex. **a** In vitro transcription by HelaScribe extracts in the presence of competitor MVI domains at 25 μM, unless stated otherwise. Sites B and C refer to _C_CBD_ΔSiteB_ and _C_CBD_ΔSiteC_, respectively. TIP refers to the control reaction performed in the presence of 25 μM of the MVI inhibitor (TIP). Samples were normalised to the control sample in Fig. 6f (error bars represent SEM from five independent experiments **p < 0.001 by two-tailed *t*-test). See Supplementary Fig. 8c for control experiments. **b** In vitro transcription following antibody depletion and rescue using recombinant MVI (1 μM), NDP52 (5 μM) and F-actin (5 μM), as described in Methods (error bars represent SEM from five independent experiments **p < 0.001 by two-tailed *t*-test). **c** Co-immunoprecipitation of MVI and RNAPII, using an antibody against MVI to immunoprecipitate RNAPII from HeLa cells. Immunoprecipitation does not occur in HeLa cells subjected to siRNA knockdown of MVI. **d** Western blot against RNAPII following isolation from nuclear extracts by MVI constructs. Loading controls for the recombinant proteins is shown in Supplementary Fig. 9. **e** Working model of MVI in transcription elongation. MVI is bound to partner and/or DNA at the C terminus and RNAPII through actin at the N terminus

Building upon our knowledge of which MVI domains bind DNA, we explored how these domains correspond to coupling MVI to the RNAPII complex. We employed a competition assay to displace the native protein using individual recombinant domains and then performed in vitro transcription. The CBD alone was able to displace MVI, leading to a decrease in transcription in a CBD concentration dependent manner (Fig. 7a). This effect was specific to the CBD and required the DNA binding ability within the domain to fully perturb the reaction. In addition, mutation of the RRL-motif revealed that successful competition is likely to be driven by partner association at this location. This was further supported by the lack of inhibition by the LI-CBD.

Our observation of the CBD competing off the MVI, leading to a decrease in transcription, indicated that the FL-MVI, is required for transcription. We tested whether the isolated MVI motor domain can function as a competitor. Indeed, the motor is a competitor (Fig. 7a), suggesting that MVI forms a bi-polar interaction with the RNAPII complex, which is vital for transcription. The importance of the motor domain was further confirmed when we used the MVI inhibitor 2,4,6-triiodophenol (TIP)^33^ to inhibit the MVI motor activity. This treatment resulted in a transcription level equivalent to depleting MVI.

We explored how the conformation of MVI, its motor activity and, more specifically, actin binding can affect transcription. To further reinforce our results, we performed rescue assays on antibody depleted extracts, by adding recombinant proteins. The addition of FL-MVI led to a slight rescue in transcription (Fig. 7b). However, when in combination with NDP52, there was a two-fold increase in transcript production. This observation highlights that it is an active unfolded state of MVI that participates in transcription. When we incubated both samples with F-actin, we observed no increase in transcription. In this case, while interactions through the CBD could occur, the motor was prevented from coupling to RNAPII because it was bound to F-actin. Therefore, we concluded that the actin binding site of MVI is required in transcription, possibly by coupling it to the complex through RNAPII-bound nuclear actin^31, 32^.

MVI can co-immunoprecipitate RNAPII from HeLa extracts, as shown previously^3^ (Fig. 7c). Subsequently, the recombinant MVI truncations were also tested for their ability to act as bait for RNAPII in nuclear extracts (Fig. 7c, d). Indeed, their ability to bind RNAPII was consistent to their ability to inhibit transcription. The _C_CBD showed high RNAPII binding potential, whereas the _N_CBD was weak. This suggests that the main coupling factor to the RNAPII complex is the DNA binding sites of the _C_CBD, rather than the RRL motif. However, the LI did not bind to RNAPII, confirming that the RRL is still required for the interaction. Moreover, DNA binding is only possible once MVI is unfolded which is driven by binding partner at the RRL motif.

Overall, we propose a model whereby MVI is coupled to RNAPII through both motor-actin as well as interactions of the CBD with binding partners and/or DNA. This bipartite association to the complex in an active unfolded state enables MVI to utilise its mechanical ability during transcription to help drive or anchor the complex (Fig. 7e).

### Myosin VI is coupled to gene expression by nuclear receptors

Here we have shown that MVI and NDP52 have a role in transcription. Given the overexpression of MVI in breast, ovarian and prostate cancer^5, 7, 18^, our findings indicated a possible link between MVI and the expression of genes under the regulation of hormone receptors. There is evidence of coupling MVI to the androgen receptor^34^. However, this has not been explored for other hormone receptors, nor has the consequence upon gene expression been investigated.

**Fig. 8:**
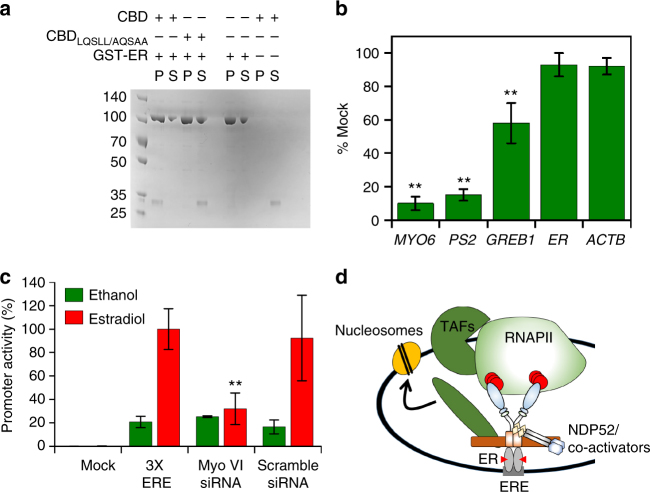
Myosin VI is linked to oestrogen signalling. **a** Pull-down of 2 μM CBD by 10 μM GST-ER. P and S represent pellet and supernatant fractions. **b** Expression of ER gene targets following siRNA knockdown of MVI in MCF-7 cells. Expression is plotted as a percentage of expression in mock cells. ER (*ESR1*) and β-actin were used to reflect global changes in transcription. **c** Luciferase reporter assay driven by the ERE promoter in MCF-7 cells. Estradiol led to a 5-fold increase in promoter activity. siRNA knockdown of MVI led to a 3-fold reduction in activity. Ethanol was used as a carrier control for the experiments (error bars represent SEM from three independent experiments **p < 0.001 by two-tailed *t*-test). **d** Working model of MVI recruitment by the ER. MVI binds to the ER and then gets activated by NDP52 (or CoCoA), which enables binding to RNAPII through actin. Association with NDP52, or CoCoA, ties MVI with the general transcription co-activators to initiate recruitment of RNAPII

To address such an intriguing possibility, we inspected the C terminus of MVI and identified an LxxLL nuclear receptor binding motif. In addition, as shown in Fig. 8a, pull-down assays with recombinant proteins revealed that there is a direct interaction between the CBD and the oestrogen receptor (ER). Moreover, mutation of this LxxLL motif abolished binding.

To explore the role of MVI in transcription of ER regulated genes, we switched to using ER positive MCF7 cells and monitored the expression of the ER target genes *PS2* and *GREB1*. Knockdown of MVI resulted in a specific 85% and 40% decrease in expression, respectively (Fig. 8b and Supplementary Fig. 8d), thereby suggesting a link between receptor binding and MVI’s role in transcription. It is important to note that MVI knockdown does not lead to a general decrease in expression. To explore this relationship in a more general context, we also performed luciferase reporter assays driven by the ERE (ER response element) reporter. As shown in Fig. 8c, the stimulatory effect of estradiol is impeded upon a knockdown of MVI. We therefore suggest MVI may have the potential to regulate genes under ERE control.

## Discussion

Here we have explored the function and regulation of nuclear MVI. The association of MVI with RNAPII is dependent upon its ability to bind DNA, which is regulated by putative transcription co-activator NDP52. NDP52 alters the MVI oligomeric and mechanical properties. We suggest MVI may function as an auxiliary motor in transcription, with a bipartite coupling to the RNAPII complex through NDP52-DNA, as well as RNAPII-bound actin. Finally, MVI interacts with nuclear receptors for specific gene targeting (Fig. 8d). These processes are structurally confined to the nuclear localising NI isoform, frequently found in cancer cells.

We have shown how a myosin can directly bind DNA. This occurs through two sites on the CBD that are inaccessible in FL-MVI. We observed MVI adopting a folded conformation consistent with previous analysis^24, 26, 35^. Importantly, we have provided evidence this occurs in vivo using FLIM. Here we present a model whereby binding partners can unfold MVI to expose these sites. Unfortunately, the direct DNA binding by NDP52 prevented the direct measurement of a partner regulating this process. Nevertheless, we have revealed a mechanism whereby NDP52 can unfold MVI. The non-specific selectivity for substrate and the relatively weak binding affinity for nucleic acids suggests that DNA binding is likely to be driven through partners and their abilities to bind specific DNA targets.

Here we have defined that activation of MVI occurs through unfolding to enable both its motility and mechanical work. However, no change in ATPase activity has been observed. It is here that the role of Ca^2+^-calmodulin are likely to participate to fine-tune the motor activity in order to increase or reduce the ATPase activity, as required.

Our regulatory mechanism also enabled us to look into MVI oligomerisation. Here we report that MVI can be both a monomer and dimer dependent upon binding partner, with the latter controlled by the innate ability of the protein to dimerise. Our model is in agreement with the previous conclusions^28, 36^, where it was proposed that MVI adopts a folded conformation and cargo bridges two motors, with the close proximity leading to internal dimerisation^37^. We found that NDP52 triggered this process, and it occurred by exposing the dimerisation domain through unfolding. We suggest dimerisation occurs within the _N_MVI_TAIL_ (amino acids 814–1060), which is in agreement to the model proposed by Mukherjea^8^ where the authors proposed dimerisation occurring distal to the 3HB between residues 917–942. Moreover, a later study confirmed this site was necessary for MVI to function in vivo^38^ and the SAH domain (residues 842–978) was redundant.

The dissociation constant for dimerisation within the _N_MVI_TAIL_ is essentially identical to the MVI_TAIL_ in the presence of NDP52, where the latter must be initially unfolded. This assessment is in agreement with molecular dynamics simulations, which showed self-association within this region of the MVI_TAIL_^39^. Moreover, our data agree with the experimental evidence from Spink^26^, whereby the tail was monomeric at 1 μM. However, at higher concentrations we observed dimerisation (Fig. 5a and Supplementary Table 4).

We have also explored the nuclear role of NDP52 and linked the protein to both MVI and RNAPII. Many of our results suggest NDP52 participates in transcription: (i) its DNA-binding ability, (ii) its high homology to transcription co-activator CoCoA, (iii) its colocalisation with RNAPII, (iv) its binding to the RNAPII complex and (v) the observation that its depletion decreases mRNA yield in vitro. We suggest NDP52 can function similar to CoCoA. Likewise, we revealed that CoCoA is also a MVI partner. Further investigation is now required to define the nuclear role of NDP52.

MVI was previously linked to transcription initiation and elongation, whereby depletion reduced transcription^3^. We have expanded upon this study, firstly by dissecting its effect in a quantitative manner and secondly, by determining the domains of MVI responsible for its interaction with RNAPII. We have revealed that the CBD is associated with the RNAPII complex, requiring both the RRL motif and the DNA-binding sites. We suggest that NDP52, and/or CoCoA, bind to the RRL motif to unfold MVI.

Importantly, RNAPII interactions are not limited to the C terminus of MVI. The motor domain is likely to associate with RNAPII through actin. Due to the presence of actin on the RNAPII complex^31, 32^, it is highly likely for MVI to bind this actin population. Whilst the oligomeric state of this actin remains unknown, it has been suggested that it consists of short polymers^40^, thereby providing several binding interfaces across the complex. The importance of the MVI–actin interaction is more relevant when we used the TIP inhibitor to target the motor activity. Inhibition resulted in a transcription decrease equivalent to depletion. Therefore, we conclude that MVI is an active motor in transcription.

Our finding that suggests a bipartite association between MVI and the RNAPII complex would enable mechanical work to be performed, whereby MVI could either hold the complex in situ, or act as an auxiliary motor driving RNAPII (Fig. 7e). MVI can perform both functions, switching between them depending on the force exerted on it^41^. We speculate that MVI could help propel the RNAPII complex, but should the complex encounter a stall, it could exert a load upon MVI, triggering a switch to a ‘tether mode’ until transcription begins again. Indeed, MVI has been associated with transcription pause-restart^21^.

MVI has previously been linked to the androgen receptor (AR), with evidence for a direct interaction between the two^34^. We identified a nuclear receptor binding motif at the C terminus of MVI which enabled ER binding, suggesting a wider link to nuclear receptors beyond the AR. A link between MVI and nuclear receptors is of particular importance, given their role in cell proliferation, which is reduced after MVI knockdown^18, 20^. We found that MVI knockdown led to a decreased expression of ER target genes. Moreover, luciferase reporter assays showed that MVI is involved in regulating expression from ERE promoters. Therefore, MVI may participate in the activation of ER-driven gene expression (Fig. 8d), which drives tumour development and maintains the tumorigenic potential of cells. A wider genomic study is required to reveal the extent of this effect.

In summary, we have revealed how the motor protein can bind DNA in a process regulated by NDP52. The binding partner releases an autoinhibited state, which also enables dimerisation. This couples MVI to the RNAPII complex, where it can function as a tethering factor and/or auxiliary motor driving transcription. Lastly, we have revealed how MVI interacts with nuclear receptors with the potential to modulate gene expression.

## Methods

### Constructs

A list of constructs and PCR primers are provided in Supplementary Tables 5 and 8, respectively. Human MVI_TAIL_ and _N_MVI_TAIL_ were generated using directional TOPO cloning in to pET151 using pEGFP-NI-MVI as a template. The CBD, _N_CBD_1060-1120_ and _C_CBD_1121-1253_ were isolated by PCR using pET151 MVI_TAIL_ as a template, restriction digested by NheI and NotI and cloned into pET28. MVI_TAIL_ (large insert) and CBD (large insert) were cloned by PCR and restriction digestion using NheI and NotI in to pET28 using pEGFP-C3-LI-MVI as a template. CBD M1062Q (large insert) was cloned in the same manner from pEGFP-C3-LI-MVI M1062Q. CBD ΔSite A was cloned as above from pEGFP-C3-NI-MVI WKSKNKKR/WASANNNR. MVI, Motor_1-814_ and Motor_1-1060_ were cloned by PCR and restriction digest (NotI and XhoI) into pFastbacHTB using pFastbacHTB-NI-GFP-MVI as template. pET28 calmodulin was cloned by PCR and restriction digestion from pFastbac1calmodulin.

Modified CBD and MVI_TAIL_ constructs were made by site-directed mutagenesis using standard Quick-Change site-directed mutagenesis protocol. All plasmids were verified by DNA sequencing.

The dual-labelled full-length myosin VI construct was generated by restriction digest of pEGFP-NI-MVI with XcmI (within MVI) and SacII (in vector) to remove the last 67 residues at the C terminus of myosin VI. A synthetic gene was produced (pMXMVI C terminus) containing the C terminus with both cut sites but mRFP was introduced prior to the SacII site. The construct was then assembled to yield FL pEGFP-NI-MVI-RFP.

### Protein expression and purification in *Escherichia coli*

Recombinant constructs were expressed in *E.coli* BL21 DE3 cells (Invitrogen) in Luria Bertani media. Proteins were purified by affinity chromatography (HisTrap FF, GE Healthcare). The purest fractions were desalted through a PD10 column (GE Healthcare) to remove imidazole before treatment with TEV protease for 4 h at 25 °C. The samples were then passed through a second HisTrap column. The cleaved protein was further purified through a Superdex 200 16/600 column (GE Healthcare). Note: TEV cleavage was not performed when the His-tag was needed for pull-down experiments.

### Protein expression using baculovirus system

Full-length myosin VI, Motor_1-814_, Motor_1-1060_ and *Xenopus* calmodulin were expressed in *Sf9* and *Sf21* (*Spodoptera frugiperda*) insect cells using the Baculovirus expression system. *Sf9* cells were cultured in suspension in sf900 media (Gibco) at 27 °C to generate the P1-3 recombinant baculovirus stocks. Finally, expression of recombinant proteins was set up by infecting *sf21* cells with the P3 viral stock in ExCell 420 media (Sigma). The cells were harvested by centrifugation for protein purification after 4 days. Prior to sonication, an additional 5 mg Calmodulin was added with 2 mM DTT. After sonication, 5 mM ATP and 10 mM MgCl_2_ were added and the solution was rotated at 4 °C for 30 min before centrifugation (20,000 × *g*, 4 °C, 30 min). Then, the cell lysate was subjected to the purification steps described above.

### Protein labelling

Proteins were transferred into 50 mM Na-phosphate (pH 6.5) using a PD10 column. Samples were then incubated with a 5-fold excess of dye for 4 h, rotating at 4 °C. Excess dye was removed using a PD10 column pre-equilibrated with 50 mM Na-Phosphate, 150 mM NaCl and 1 mM DTT. Labelling efficiency was calculated based on the absorbance at 280 nm and the absorbance maximum of the dye. Typical efficiency was 90%, whereby the less than complete labelling was taken as an indicator for a single dye per protein. This was tested for isolated preparations in mass spectroscopy, which revealed both an unlabelled and single labelled population.

### Cell culture and transfection

HeLa (ECACC 93021013) and MCF7 (ECACC 86012803) cells were cultured at 37 °C and 5% CO_2_, in Gibco MEM Alpha medium with GlutaMAX (no nucleosides), supplemented with 10% Fetal Bovine Serum (Gibco), 100 units per ml penicillin and 100 µg ml^−1^ streptomycin (Gibco). For the transient expression of MVI isoforms, HeLa cells grown on glass coverslips were transfected with EGFP-NI-MVI, EGFP-LI-MVI and EGFP-LI-MVI(M1062Q) constructs using Lipofectamine 2000 (Invitrogen), following the manufacturer’s instructions. At 72 h post transfection, cells were subjected to nuclear staining, fixed and analysed or subjected to indirect immunofluorescence (see below). For MVI knock-down experiments, MCF7 monolayers, seeded to 30–50% confluency, were transfected with human myosin VI siRNA duplex (5′-GGUUUAGGUGUUAAUGAAGtt-3′) (Ambion) or AllStars Negative Control siRNA duplex (Qiagen) at a concentration of 50 nM, using Lipofectamine 2000 (Invitrogen), according to the manufacturer’s guidelines. Cells were harvested after 48 h for immunoblot and RT-qPCR analysis.

### Nuclei isolation

The nuclei isolation protocol was adapted from the Collas Lab protocol^42^. HeLa cells were washed once with ice-cold PBS, then washed in ice-cold Hypotonic Buffer N (10 mM Hepes pH 7.5, 2 mM MgCl_2_, 25 mM KCl supplemented with 1 mM PMSF,1 mM DTT and 1x Halt Protease Inhibitor Cocktail (Thermo Fisher Scientific)). Cells well then re-suspended in ice-cold hypotonic buffer N and incubated for 1 h on ice. Cells were then homogenised on ice with a glass Dounce homogeniser (Wheaton). Cell lysate was supplemented with 2 M sucrose solution and mixed well by inversion before centrifugation. The supernatant which corresponded to the cytoplasmic fraction, was aliquoted and stored at −80 °C. The pellet, which corresponded to isolated nuclei, was further cleaned by washing in ice-cold buffer N (10 mM Hepes pH 7.5, 2 mM MgCl_2_, 25 mM KCl, 250 mM sucrose, supplemented with 1 mM PMSF, 1 mM DTT and 1x Halt Protease Inhibitor Cocktail). The nuclei pellet was re-suspended either in ice-cold Buffer N and used immediately or in freezing medium (70% glycerol in buffer N), to yield a concentration of 4 × 10^6^ nuclei per ml. Nuclei were aliquoted on ice and stored at −80 °C.

### Nuclear staining and immunofluorescence

Purified fresh or defrosted nuclei were immobilised on Poly-d-lysine (MW 70,000–150,000, Sigma)-coated glass coverslips (ThermoFisher) by 30 min incubation at 37 °C. Glass coverslips were coated with 0.1 mg ml^−1^ Poly-d-lysine solution in H_2_O, for 30 min, at room temperature, washed and dried. Immobilised nuclei were stained with 1 μg ml^−1^ Hoechst 33342 (ThermoFisher) and/or 5 μl ml^−1^ Vybrant® DiD Cell-Labeling Solution (ThermoFisher) in Buffer N. Transfected and non-transfected HeLa cells grown on glass coverslips were incubated for 10 min at 37 ^o^C with 1 μg ml^−1^ Hoechst 33342 in growth medium. Stained cells or nuclei were fixed for 15 min at room temperature in 4% (w/v) paraformaldehyde (PFA) and residual PFA was quenched for 15 min with 50 mM ammonium chloride. All subsequent steps were performed at room temperature. Cells or nuclei were permeabilised and simultaneously blocked for 15 min with 0.1 % (v/v) Triton X-100 and 2 % (w/v) BSA in TBS. Cells or nuclei were then immune-stained against the endogenous proteins by 1 h incubation with the indicated primary and subsequently the appropriate fluorophore-conjugated secondary antibody (details below), both diluted in 2% (w/v) BSA in TBS. The following antibodies were used at the indicated dilutions: Rabbit anti-MVI (1:200, Atlas-Sigma HPA0354863-100UL), mouse-NDP52 (1:200, Abcam Ab124372), rabbit anti-RNAPII phospho S5 (1:500, Abcam Ab5131), donkey anti-mouse Alexa Fluor 647-conjugated (1:500, Abcam Ab150103) and donkey anti-rabbit Alexa Fluor 555-conjugated antibody (1:500, Abcam Ab150074). For actin staining, fixed and permeabilised cells or nuclei were stained prior to immunofluorescence with 165 nM Rhodamine-Phalloidin (ThermoFisher) for 20 min. Coverslips were mounted on microscope slides with Mowiol (10% (w/v) Mowiol 4–88, 25% (w/v) glycerol, 0.2 M Tris-HCl, pH 8.5), supplemented with 2.5% (w/v) of the anti-fading reagent DABCO (Sigma).

For colocalisation analysis, ROIs were drawn around the nuclei of individual cells, or around whole cells in the case of Fig. 4g and Supplementary Fig. 5c (5 cells and 5 stacks per cell). Pearson’s coefficients were obtained with the JACoP plugin^43^ for ImageJ.

### Immunoblot analysis

The total protein concentration of the cytoplasmic and nuclei fraction was determined by Bradford Assay (Sigma) following the manufacturer’s instructions. Nuclei, cytoplasmic fractions and cell lysates were heat-denatured and resolved by SDS-PAGE. The membrane was probed against the endogenous proteins by incubation with the indicated rabbit polyclonal primary and subsequently a goat anti-rabbit antibody coupled to horseradish peroxidase (1:15,000 Abcam Ab6721). The following primary antibodies were used at the indicated dilutions: Rabbit anti-MVI (1: 500, Atlas-Sigma HPA0354863-100UL), Rabbit anti-NDP52 (1:2000, GeneTex GTX115378), Rabbit anti-alpha tubulin (1:1000, Santa Cruz sc5286), Rabbit anti-Lamin B (1:1000, Abcam ab16048 and rabbit anti-RNAP II phospho S5 (1:1000, Abcam Ab5131). The bands were visualised using the ECL Western Blotting Detection Reagents (Invitrogen) and the images were taken using Syngene GBox system. Images were processed in ImageJ. Uncropped blots are shown in Supplementary Figs. 9 and 10.

### Co-immunoprecipitation

Immunoprecipitation was performed using rabbit anti-myosin VI (Sigma), or rabbit anti-NDP52 (Genetex). Antibodies were bound to Protein A and G dynabeads, respectively, according to the manufacturer’s instructions. Immunoprecipitation was performed from total protein extract for 2 h in a buffer containing 50 mM Tris-HCl, 150 mM NaCl, 1 mM DTT, 1 mM PMSF and 1% Triton X-100. Samples were subjected to immunoblot for RNAPII, as described above. Where samples were compared between conditions, equal total protein concentrations were used.

### Imaging

Cells and nuclei were visualised using Olympus IX71 microscope with PlanApo 100xOTIRFM-SP 1.49 NA lens mounted on a PIFOC z-axis focus drive (Physik Instrumente, Karlsruhe, Germany), and illuminated with an automated 300 W Xenon light source (Sutter, Novato, CA) with appropriate filters (Chroma, Bellows Falls, VT). Images were acquired using a QuantEM (Photometrics) EMCCD camera, controlled by the Metamorph software (Molecular Devices). The whole volume of cells and nuclei was imaged by acquiring images at z-steps of 200 nm. Using the Hoechst 33342 staining, the lower and upper sections at which the nucleus was in focus were defined. Unless stated otherwise, images presented here correspond to a middle section between these two limits, which ensures imaging within the nucleus. Images were deconvolved with the Autoquant X software applying blind deconvolution and analysed by ImageJ.

### FLIM sample preparation and data acquisition

Transfections were performed as above with the exception that 1 mg ml^−1^ sodium borohydride (Sigma) was used for quenching. All time correlated single photon counting mode (TCPSC) images were acquired using the custom-built multifocal multiphoton fluorescence lifetime imaging system (MM-FLIM) as described previously^44^. In brief, light generated from a Chameleon Ultra II Ti:Sapphire laser source (Coherent Inc.) is coupled with a spatial light modulator (SLM) to generate a uniform 8 × 8 array of beamlets. This beamlet array is then relayed through a set of galvanometer scanners (providing *x*–*y* raster scanning capability) onto the back-pupil plane of a 40 × 1.3 N.A. Plan Fluor oil objective (Nikon) where it is projected onto the sample. The two-photon generated fluorescence is collected and descanned where it is directed with a dichroic mirror and focused onto the Megaframe SPAD array using a 10 × 0.3 N.A. Plan Fluor air objective (Nikon).

For each individual image acquisition, the system processed 64 × 64 data points for 8 × 8 detectors producing 512 × 512 pixel images. Lifetime data was acquired operating the Megaframe camera in TCPSC mode. In TCSPC mode, on-pixel TDCs generate raw time-correlated data, which are stored and then post-processed offline to generate an image. Full details on TCSPC data acquisition can be found in ref. ^44^. Once processed, these data are saved and then subsequently analysed using TRI2 lifetime analysis software^45^.

### RNA extraction and RT-qPCR

RNA from MCF7 cells transfected with MVI or siRNA and negative control duplexes was extracted using Gene Jet RNA purification kit (Thermo scientific) according to the manufacturer’s protocol. The RNA concentration was measured using Geneflow Nanophotometer and RT-qPCR was performed with one-step QuantiFast SYBR Green qPCR kit (Qiagen) using 50 ng of RNA in each sample. A list of qPCR primers is given in Supplementary Table 6.

### Dual-luciferase assay

MCF7 cells were seeded in a 24-well plate at a density of 1.0 × 10^5^cells per well in no phenol red MEM media (Gibco) with 5% double charcoal stripped FCS (First Link UK) and 1% Penicillin-Streptomycin. After 48 h of starvation, the cells were co-transfected with 3X-ERE-TATA-Luc expression plasmid (400 ng per well), RL-CMV plasmid (400 ng per well) and human myosin VI siRNA duplex (5′-GGUUUAGGUGUUAAUGAAGtt-3′) (Ambion) or AllStars Negative Control siRNA duplex (Qiagen) at a concentration of 50 nM, using Lipofectamine 2000 (Invitrogen), according to the manufacturer’s guidelines. In the control wells, cells were co-transfected with 3X-ERE-TATA-Luc and RL-CMV expression plasmids only. After 24 h of transfection, cells were treated with 10 nM of Estradiol or vehicle control and the luciferase activity was measured the following day using Dual-Glo luciferase reporter assay kit (Promega). Levels of firefly luciferase were standardised to those of Renilla. All samples were measured in triplicates.

### DNA substrates

Labelled and unlabelled oligonucleotides were purchased from Sigma-Aldrich. To form duplex DNA substrates, oligonucleotides were mixed at equimolar concentrations at either 50 μM in water or a buffer containing 50 mM Tris.HCl at pH 7.5, 150 mM NaCl, and 3 mM MgCl_2_. A list of DNA substrates is given in Supplementary Table 7.

### In vitro transcription

The DNA template was the pEGFP-C3 linearised plasmid containing the CMV promoter that would generate a 130-base run-off transcript. The HelaScribe (Promega) reactions were performed in triplicates, through two independent experiments, according to the manufacturer’s instructions. The reactions were performed for 60 min at 25 °C.

Reactions were also performed following pre-clearance with the stated antibodies. Protein G or A Dynabeads (Invitrogen) were prepared according to the manufacturer’s instructions before being loaded with 4 μg antibody. Samples were incubated for 30 min on ice and beads were extracted immediately before performing the transcription reaction.

For quantification, mRNA was purified using Gene Jet RNA purification kit (Thermo Scientific) according to the manufacturer’s protocol and RT-qPCR was performed with one-step QuantiFast SYBR Green qPCR kit (Qiagen).

### Preparation of liposomes

Mixed brain liposomes—Folch fraction 1 (Sigma) were re-suspended in 20 mM HEPES (pH 7.4), 150 mM NaCl and 1 mM DTT to a final concentration of 1 mg ml^−1^. The mixture was then extruded using a 100 nm filter. Liposomes were stored at 4 °C and used within 4 days.

### Actin-pelleting assay

The assay was modified from Morriswood et al. Full length MVI was incubated with the tail construct at the specified concentrations in reaction buffer (150 mM NaCl, 50 mM Tris HCl (pH 7.5), 1 mM MgCl_2_ and 1 mM DTT), for 30 min at 4 °C. 5 μM F-actin (from M. Geeves) was added to the mixture before centrifugation at 190,000 × *g* for 15 min at 4 °C. The pellet was re-suspended in 50 μl reaction buffer and samples were analysed by SDS-PAGE and densitometry using ImageJ.

### Protein–protein and protein–DNA isolation

*His-tagged protein–protein pull downs*. 10 μM His-tagged bait protein was bound to His Mag Sepharose Ni beads (GE Healthcare) according to the manufacturer’s instructions. Samples were incubated in 50 mM Tris-HCl (pH 7.5), 500 mM NaCl and 40 mM imidazole and 1 mM DTT for 30 min shaking (400 rpm) at 25 °C. Beads were isolated and samples volumes were normalised with NuPAGE Sample buffer before SDS-PAGE.

*Pull-down from nuclear extract*. His-tagged bait protein was prepared as above. Frozen nuclei aliquots were defrosted as described above and re-suspended in 50 mM Tris-HCl (pH 7.5), 500 mM NaCl and 40 mM imidazole, 1 mM DTT and 1% Triton X. Nuclei were incubated on ice for 15 min for lysis to occur. Samples were then incubated for 30 min as above before proceeding with immunoblot analysis.

*GST-tagged protein–protein*. 10 μM GST-tagged bait protein was bound to glutathione sepharose resin (GE Healthcare) according to the manufacturer’s instructions. Samples were incubated in PBS supplemented with 1 mM DTT, shaking at 25 °C for 30 min. Beads were isolated by centrifugation (1000 × *g* for 5 min) and samples volumes were equalised with NuPAGE sample buffer before SDS-PAGE.

*DNA protein*. A single biotin labelled 500 bp DNA substrate was generated by PCR with 5′ biotin-TEG primer using pSG1365 as a template. Free biotin primers were removed using a QiaQuick PCR purification kit (Qiagen). 1 mg Streptavidin Dynabeads (M-280) (Invitrogen) were washed three times in wash buffer (10 mM Tris-HCl pH 7.5, 1 mM EDTA and 2 M NaCl). 1 μg DNA was added in an equal volume to bring the final NaCl concentration to 1 M and incubated for 15 min at room temperature. Beads were washed three times in wash buffer before re-suspension in binding buffer (50 mM Tris-HCl pH 7.5, 3 mM MgCl_2_, 1 mM DTT and 50 mM NaCl). Protein was added to the beads and incubated shaking (400 rpm) for 30 min, at 25 °C. Beads were extracted and samples volumes were normalised with NuPAGE sample buffer before SDS-PAGE.

### Gliding filament assays

Motility assays were performed at 30 °C. Antibody immobilisation was achieved using rabbit anti-myosin VI (Atlas-Sigma HPA0354863-100UL) against the C terminus that was pre-absorbed to nitrocellulose (0.1% v/v). NDP52 immobilisation was achieved by flowing 0.1, 0.5 or 1 μM NDP52 onto nitrocellulose. Subsequently, 100 µg ml^−1^ MVI was added to the flow cells in 50 mM Tris-HCl (pH 7.5), 20 mM Imidazole, 1 mM EGTA, 5 mM MgCl_2_ and 5 mM DTT. TRITC-phalloidin-labelled actin filaments were added in the same buffer plus oxygen scavenger (5 U ml^−1^ Glucose oxidase and 800 U ml^−1^ Catalase) and 2 mM ATP. Filaments were visualised every 5 s for a total period of 500 s. Individual trajectories were tracked and velocities extracted using GMimPro (www.mashanov.uk).

### Steady-state ATPase activity of MVI

Ca^2+^-actin monomers were converted to Mg^2+^-actin with 0.2 mM EGTA and 50 μM MgCl_2_ before polymerising by dialysis into 20 mM Tris-HCl (pH 7.5), 20 mM imidazole (pH 7.4), 25 mM NaCl and 1 mM DTT. A 1.1 molar equivalent of phalloidin (Sigma) was used to stabilise actin filaments.

Steady-state ATPase activities were measured at 25 °C in KMg50 buffer (50 mM KCl, 1 mM MgCl_2_, 1 mM EGTA, 1 mM DTT, and 10 mM imidazole, pH 7.0). Supplemented with the NADH-coupled assay components, 0.2 mM NADH, 2 mM phosphoenolpyruvate, 3.3 U ml^−1^ lactate dehydrogenase, 2.3 U ml^−1^ pyruvate kinase and various actin concentrations (0–30 μM). The final [Mg.ATP] was 5 mM and MVI concentration was 100–300 nM. The assay was started by the addition of MVI. The change in absorption at OD_340_ nm was followed for 5 min. The *k*_cat_ and *K*_actin_ values were determined by fitting the data .

$${\rm{Rate}} = {V_{\rm{o}}} + \left( {\frac{{{k_{{\rm{cat}}}}[{\rm{Actin}}]}}{{{K_{{\rm{actin}}}} + [{\rm{Actin}}]}}} \right)$$

*V*_o_ is the basal ATPase activity of MVI, *k*_cat_ is the maximum actin-activated ATPase rate and *K*_actin_ is the concentration of actin needed to reach half maximal ATPase activity.

### Stopped flow measurements

A HiTech SF61DX2 apparatus (TgK Scientific Ltd, Bradford-on-Avon, UK) with a mercury-xenon light source and HiTech Kinetic Studio 2 software was used^46–50^. Anisotropy was measured with the instrument in the ‘T’ format, allowing simultaneous acquisition of horizontal (*I*_//_) and perpendicular (*I*_⊥_) components. This enabled anisotropy (*I*_//_ − *I*_⊥_)/(*I*_//_ + 2*I*_⊥_), and intensity (*I*_//_ + 2*I*_⊥_) to be calculated from the same set of data. The G-factor was accounted for by normalising the detectors prior to performing measurements. Excitation was at 495 nm with emission through a 515 nm cut-off filter (Schott Glass). In all experiments, the quoted concentrations are those in the mixing chamber, except when stated. All experiments were performed at 25 °C in 50 mM Tris-HCl, 150 mM NaCl, 1 mM DTT and 3 mM MgCl_2_. The dead time of the stopped-flow instrument was ~2 ms: during this initial time no change in fluorescence can be observed.

### Titration measurements

All reactions were performed at 25 °C in a buffer containing 50 mM Tris·HCl (pH 7.5), 150 mM sodium chloride and 1 mM DTT in a final volume of 100 μl. Measurements were performed using a ClarioStar Plate Reader (BMG Labtech) with the exception of the tryptophan measurements which were performed in a Cary Spectrophotometer (Varian).

Intensity measurements were performed at the following wavelengths: Tryptophan (ex. 295 nm), GFP (ex. 490 nm), RFP (ex. 585 nm), FITC (ex. 490 nm), Alexa Fluor 555 (ex. 555 nm). FITC to Alexa Fluor 555 FRET measurements were performed using the following wavelengths ex. 470 nm and em. 575 nm. Anisotropy was measured with the instrument in the T format, allowing simultaneous acquisition of parallel (*I*_//_) and perpendicular (*I*_┴_) components using BMG filter-sets for fluorescein (Ex. 482/16-10, Dichroic LP504 and em. 530/-40).

### Analysis of kinetic data

For fluorescence anisotropy titrations: anisotropy was calculated, as described below, based upon established procedures^46, 47, 51, 52^.

Total fluorescence intensity (*F*_t_) is given by:

$${F_{\rm{t}}} = {\sum} {{c_i}{F_i}}$$

Total anisotropy (*A*_t_) is given by:

$${A_{\rm{t}}} = \frac{{{\sum} {{c_i}{F_i}{A_i}} }}{{{F_{\rm{t}}}}}$$

Where *c*_*i*_ is the concentration of species *i*, *F*_*i*_ is the fluorescence intensity per unit of concentration and *A*_*i*_ is the anisotropy. This is calculated from the parallel and perpendicular fluorescence intensity (*I*) in relation to the plane of excitation by:

$${A_i} = \frac{{{I_{{\rm{parallel}}}} - {I_{{\rm{perpendicular}}}}}}{{{I_{{\rm{parallel}}}} + 2{I_{{\rm{perpendicular}}}}}}$$

As anisotropy is additive for multiple fluorescence species in solution, it is used to give a measure of their relative concentrations. For MVI (and various constructs) there are two fluorescence species, DNA and MVI.DNA. The total anisotropy can then be calculated in terms of the dissociation constant (*K*_d_) for the MVI.DNA complex:

$${A_{\rm{t}}} = \frac{{{A_{{\rm{DNA}}}}\left( {{{[{\rm{DNA}}]}_{\rm{t}}} - [{\rm{MVI}}{\rm{.DNA}}]} \right) + {A_{{\rm{MVI}}{\rm{.DNA}}}}Q[{\rm{MVI}}{\rm{.DNA}}]}}{{{{[{\rm{DNA}}]}_{\rm{t}}} - [{\rm{MVI}}{\rm{.DNA}}] + Q[{\rm{MVI}}{\rm{.DNA}}]}}$$

where

$${[{\rm{MVI}}.{\rm{DNA}}] = \frac{{\left( {{{[{\rm{MVI}}]}_{\rm{t}}} + {{[{\rm{DNA}}]}_{\rm{t}}} + {K_{\rm{d}}}} \right) - \sqrt {{{\left( {{{\rm{[MVI]}}}_{\rm{t}}} + {[{\rm{DNA}}]_{\rm{t}}} + {{K_{\rm{d}}}} \right)}^2} - 4{{[{\rm{MVI}}]}_{\rm{t}}}{{[{\rm{DNA}}]}_{\rm{t}}}}}}{2}}$$

And where [MVI]_t_ and [DNA]_t_ are the total concentrations for each reactant. [MVI.DNA] is the concentration of the protein-bound DNA complex. *Q* is the fluorescence intensity of MVI.DNA relative to DNA. The anisotropy data were fitted to obtain dissociation constants based on the above equations using GraFit fitting software^53^.

For the FRET titrations: the 575 nm intensity data were corrected for the increase in intensity due to a small direct excitation. This background signal was subtracted from the dataset to leave the FRET values. The titration curves for the MVI_TAIL_ interactions were fitting to a binding quadratic equation:

$${[{\rm{Complex}}] = \frac{{\left( {{{[{\rm{FITC}}]}_{\rm{t}}} + {{[{\rm{AF}}555]}_{\rm{t}}} + {K_{\rm{d}}}} \right) - \sqrt {{{\left( {{{[{\rm{FITC}}]}_{\rm{t}}} + {{[{\rm{AF}}555]}_{\rm{t}}} + {K_{\rm{d}}}} \right)}^2} - 4{{[{\rm{FITC}}]}_{\rm{t}}}{{[{\rm{AF}}555]}_{\rm{t}}}} }}{2}}$$

For lipid titrations: curves were fitted to a 1 site plus background equation:

$$[{\rm{Fluorescence}}] = \frac{{{\rm{Amplitude}}.[{\rm{Lipid}}]}}{{{K_{\rm{d}}} + [{\rm{Lipid}}]}} + {\rm{Background}}$$

### Data availability

The data supporting the findings of this study are available from the corresponding author on request.

## Electronic supplementary material

Supplementary Information

Description of Additional Supplementary Files

Supplementary Movie 1

Supplementary Movie 2

Supplementary Movie 3

Supplementary Movie 4

Supplementary Movie 5

Supplementary Movie 6

Supplementary Movie 7

Supplementary Movie 8
