# Supplementary material for: NDP52 activates nuclear myosin VI to enhance RNA polymerase II transcription
Source: Nat Commun. 2017 Nov 30;8:1871. doi: 10.1038/s41467-017-02050-w (PMC5707354; doi:10.1038/s41467-017-02050-w)
Supplement: Supplementary file 1 — Supplementary Information [file 41467_2017_2050_MOESM1_ESM.pdf]

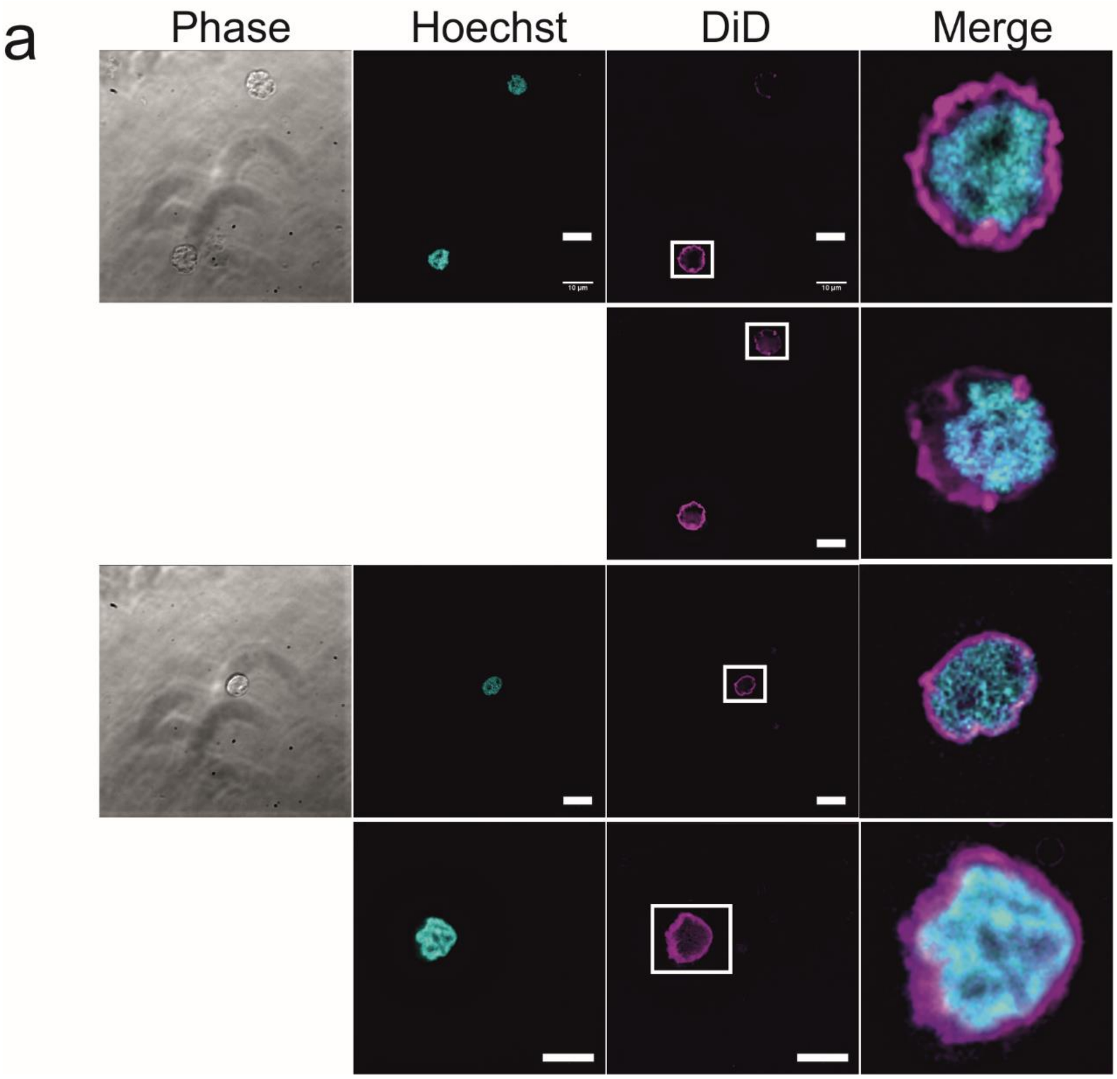

Supplementary Figure 1.

(a) Examples of purified HeLa nuclei. Nuclei were stained with the DNA dye Hoechst 33342 and the lipophilic dye DID. The staining showed that the purified nuclei appeared intact 3-D organelles, surrounded by membrane and maintaining not only their DNA content, but also preserving structures like the nucleoli (See Supplementary Movie 3 and 4). Images were acquired at the mid-point of the nucleus. Scale bar 10 μm.

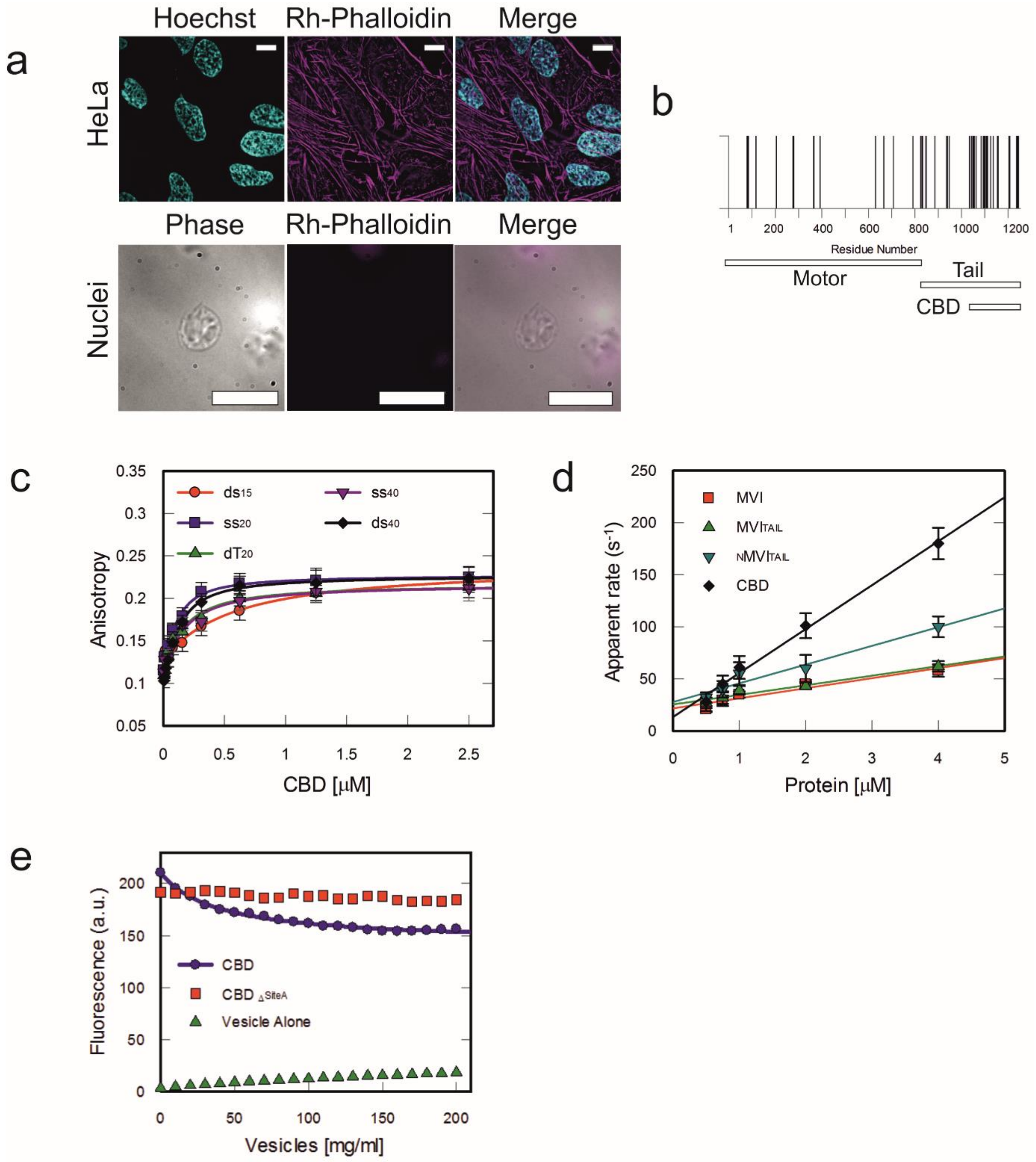

Supplementary Figure 2.

(a) Staining for filamentous actin. To assess whether the filamentous structures seen by the immunofluorescence staining of MVI were nuclear actin filaments, fixed HeLa cells and isolated nuclei were stained with Rhodamine-Phalloidin and Hoechst 3342. However, the lack of phalloidin staining in the nucleus suggested that these structures are not actin-based. Images were acquired at the mid-point of the nucleus. Scale bar 10 μm.

(b) Prediction of DNA binding residues using BindN (http://bioinformatics.k-state.edu/bindn/). Bars indicate residues with a predicted confidence greater than 70%. A full list of residues are presented in Supplementary Table 1. A majority of sites are clustered in the CBD.

(c) Fluorescence anisotropy titrations of the CBD domain against various FAM-DNA substrates (50 nM). Data fitting was performed as described in Methods. K_d: ds_{15} 500 +/- 45 nM, ss_{20} 110 +/- 35 nM, dT_{20} 120 +/- 25 nM, ss_{40} 120 +/- 35 nM and ds_{40} 100 +/- 20 nM. Data were averaged from three independent experiments.

(d) Fluorescence anisotropy was measured in stopped flow pre-steady-state measurements. MVI domains were rapidly mixed against 50 nM 40 bp FAM-DNA. Traces were fitted as described in the Methods to yield the rate constants plotted in d. Association and dissociation rate constants were calculated from linear fits to the data (Supplementary Table 3). Data were averaged from three independent experiments.

(e) Titration of Folch vesicles against 1 μM CBD or $CBD_{\Delta SiteA}$. Data were fitted as described in Methods, following background subtraction of the folch auto-fluorescence. Fitting gives an indication of the affinity with K_d 39 +/- 4 mg/ml. Due to the unknown mixed composition, it was not possible to have molar concentrations. Poor binding by $CBD_{\Delta SiteA}$ means data could not be fitted to the model.

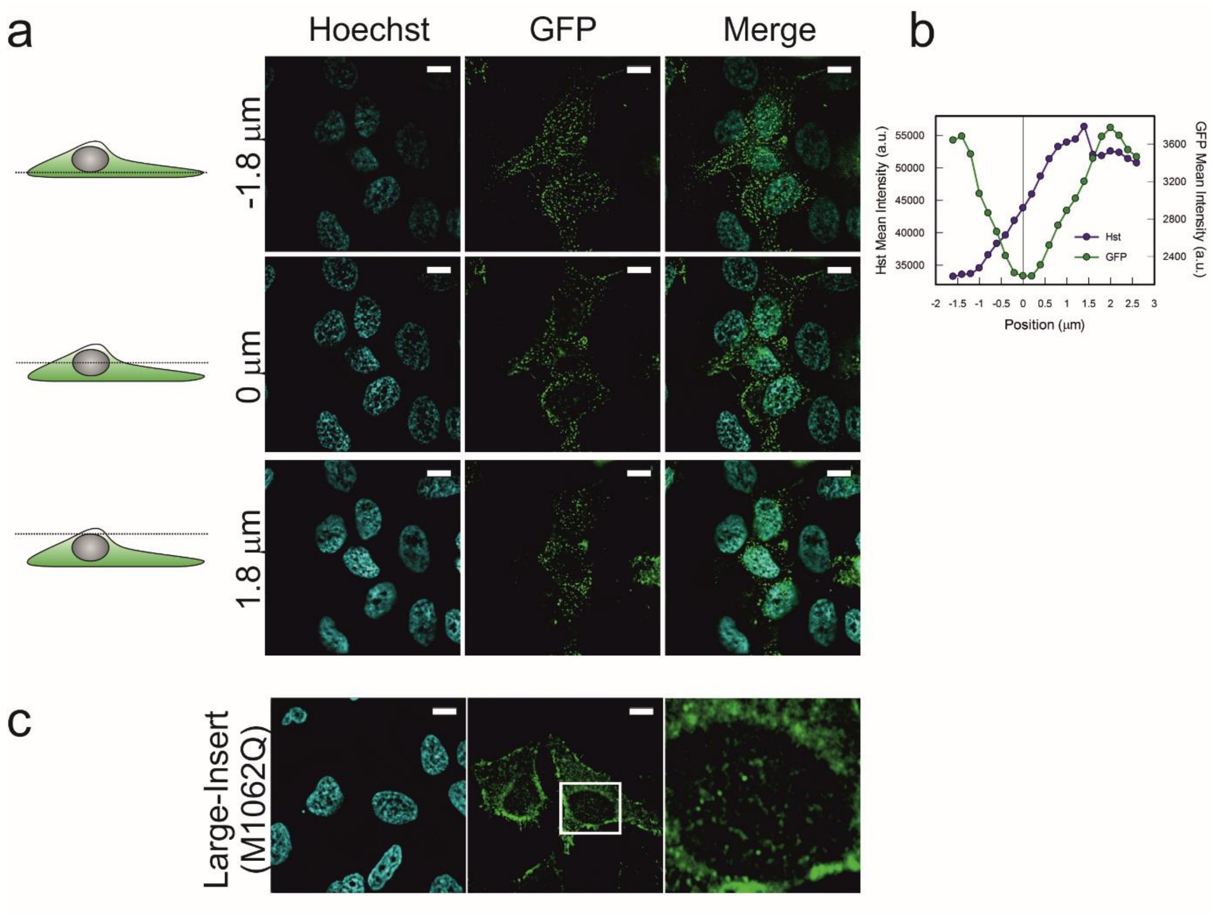

Supplementary Figure 3.

(a-b) Intracellular distribution of EGFP-LI-MVI transiently expressed in HeLa cells. The EGFP-LI-MVI localisation was restricted to the cell periphery, being excluded from the nucleus. This is highlighted by the plot of mean intensity of Hoechst (Hst) and EGFP across each image stack. The position is measured relative to the mid-point of the nucleus. The intensity was measured within a region of interest through the centre of the nucleus. Scale bar 10 μm.

(c) Intracellular distribution of EGFP-LI-MVI(M1062Q). The mutant, in which the LI helix is destabilised, displayed a similar distribution to the NI isoform. Images were acquired at the mid-point of the nucleus. Scale bar 10 μm.

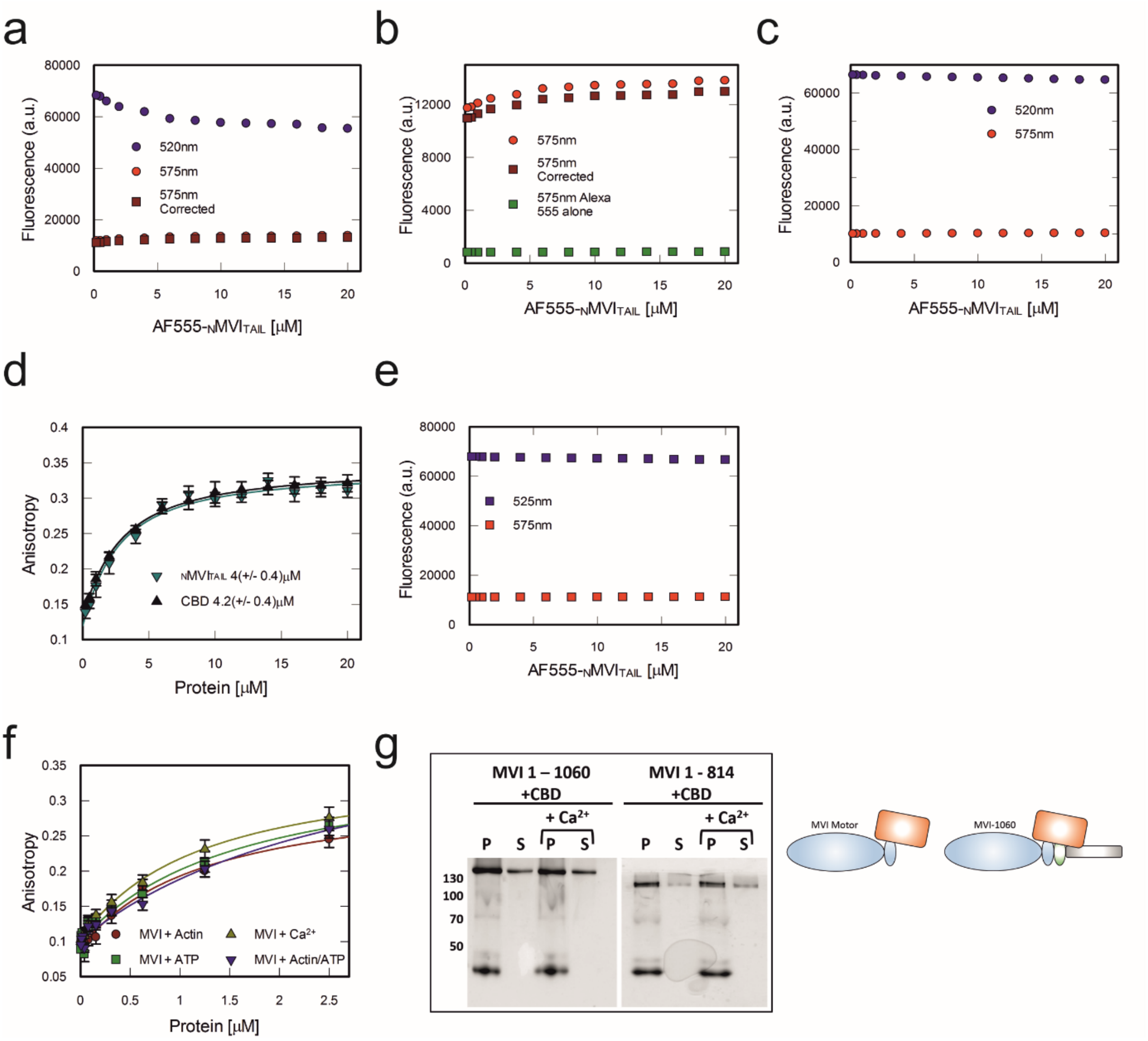

Supplementary Figure 4.

(a) Raw fluorescence intensity data for titration of $_{\mathrm{N}}$MVI$_{\mathrm{TAIL}}$ against CBD in Fig. 3a.

(b) Raw intensity data as shown in (a) but on a smaller scale.

(c) Raw fluorescence intensity data for titration of $_{\mathrm{N}}$MVI$_{\mathrm{TAIL}}$ against CBD in the presence of DNA (Blue is FITC intensity and Red is AF555 intensity).

(d) Fluorescence anisotropy titration of $_{\mathrm{N}}$MVI$_{\mathrm{TAIL}}$ against FITC-CBD and CBD against FITC-$_{\mathrm{N}}$MVI$_{\mathrm{TAIL}}$. Data were fitted as described in Methods for the DNA substrates with K_d +/- SEM.

(e) Raw fluorescence intensity data for titration of $_{\mathrm{N}}$MVI$_{\mathrm{TAIL}}$ against CBD in the presence of NDP52. (Blue is FITC intensity and Red is AF555 intensity)

(f) Fluorescence anisotropy titrations of FL MVI against a 40 bp FAM-DNA (50 nM) in the presence of F-Actin (5 μM), Ca^{2+} (1 mM), ATP (2 mM) and Actin-ATP. Data fitting

was performed as described in Methods. Data were averaged from three independent experiments.

(g) Pull-down of CBD (5 μM) by His-tagged $Motor_{1-1060}$ and $Motor_{1-814}$ both at 10 μM +/- 1 mM Ca^{2+}. P and S represent pellet and supernatant fractions, respectively. Interaction of the CBD with the motor constructs is shown in the cartoon scheme.

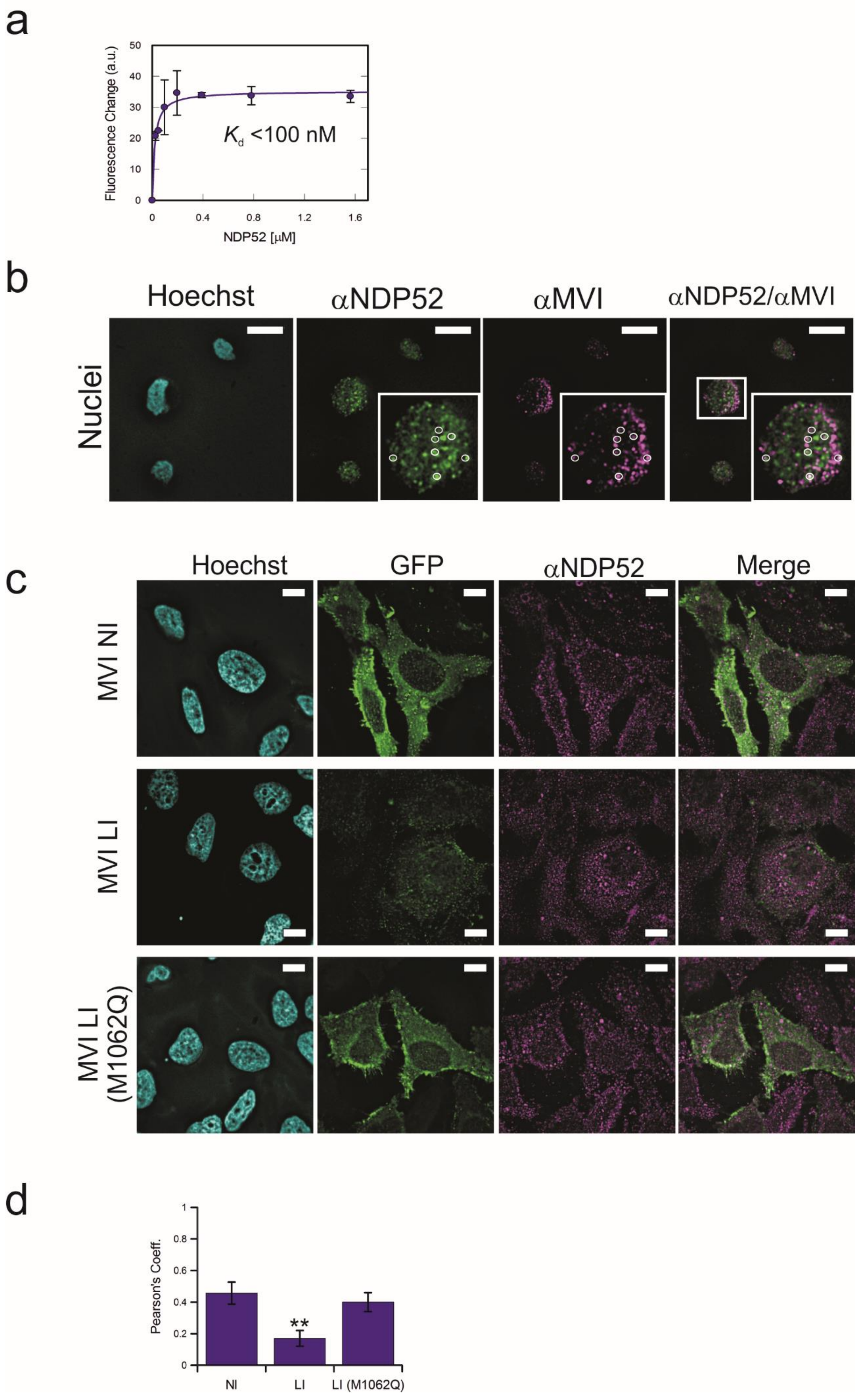

a
Fluorescence Change (a.u.)
50
40
30
20
10
0
0
0.4
0.8
1.2
1.6
NDP52 [μM]
K_d <100 nM
b
Hoechst
αNDP52
αMVI
αNDP52/αMVI
Nuclei
c
Hoechst
GFP
αNDP52
Merge
MVI NI
MVI LI
MVI LI (M1062Q)
d
Pearson's Coeff.
1
0.8
0.6
0.4
0.2
0
**
NI
LI
LI (M1062Q)

Supplementary Figure 5.

(a) Fluorescence intensity titration of NDP52 against a 40 bp FAM-DNA substrate (50 nM). Data fitting was performed as described in Methods. Data were averaged from three independent experiments. K_d: ds_{40} 80 +/- 25 nM

(b) Immunofluorescence staining against NDP52 (green), MVI (magenta) and DNA (cyan) on isolated HeLa nuclei, as performed in Fig. 1b. Insert depicts co-localising foci. Scale bar 10 μm.

(c) Representative images of transiently expressed NI-, LI- and LI (M1062Q) -EGFP-MVI in HeLa cells stained against DNA (Cyan) and immune-stained against endogenous NDP52 (magenta). Scale bar 10 μm. These images were used to assess the significance of the Pearson's coefficient regarding MVI and NDP52 co-localisation. We have established how the NI and LI (M1062Q) can form interactions with NDP52. Therefore, to assess this *in vivo* through imaging, we performed the Pearson's coefficient test (d) with the three MVI constructs. Here a region of interest was drawn around the cells and images were analysed from 5 different stacks. LI MVI shows a significantly lower co-localisation that the NI or mutant **$p < 0.001$.

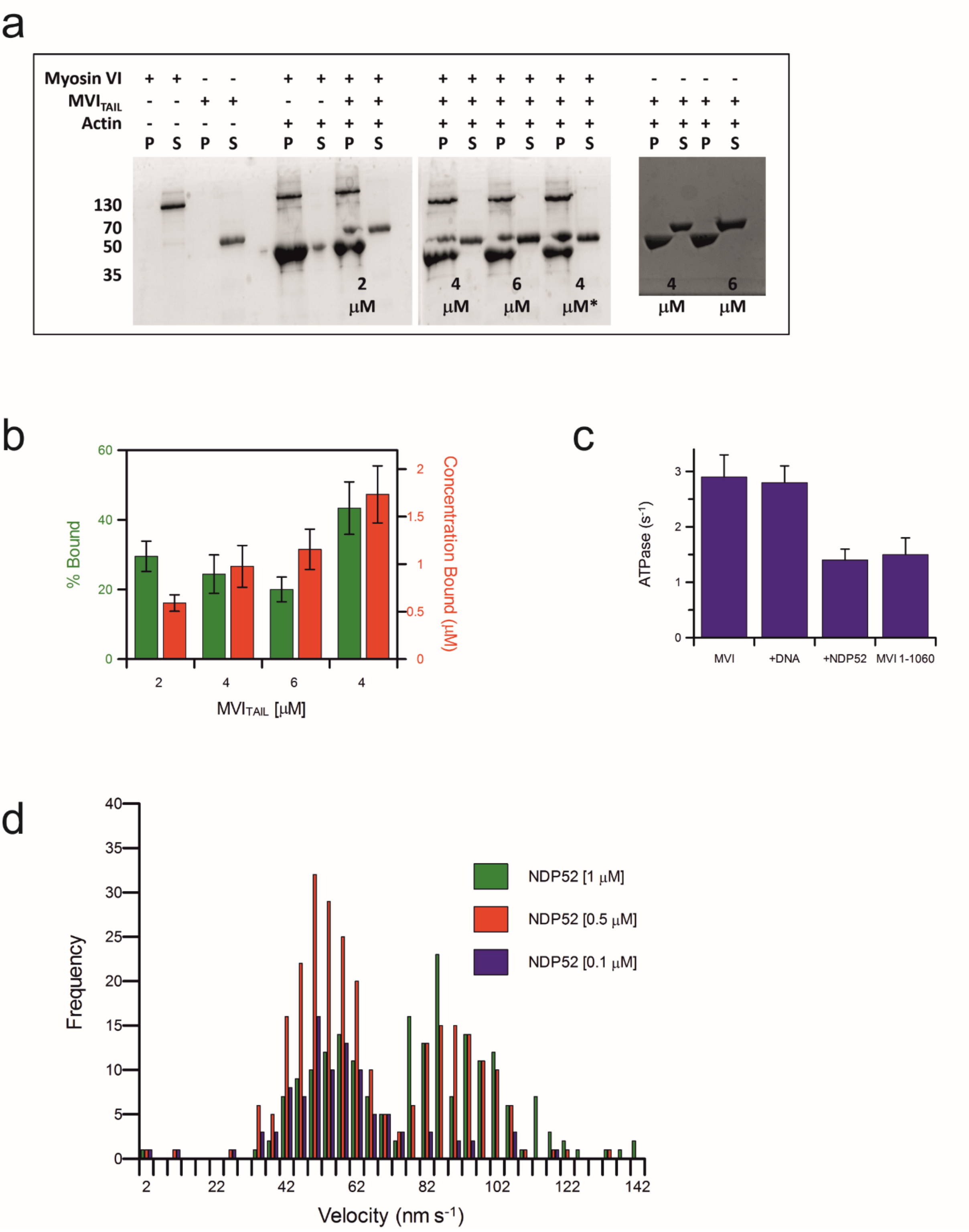

Supplementary Figure 6.

(a) Actin co-sedimentation assay. 1 μM MVI was incubated with the indicated concentrations of MVI_{TAIL} before sedimentation in the presence of 5 μM F-actin, as described in the Methods. Except for the 4 μM* condition where 2 μM MVI was used. P and S represent pellet and supernatant fractions, respectively.

(b) Gel densitometry was used to determine the relative % of MVI_{TAIL} in the actin pellet (Green bars). The % bound was then converted into the concentration of bound species (Red bars) (Error bars represent SEM).

(c) Plots of MVI or $Motor_{1-1060}$ ATPase rate constants in the presence of 5 μM DNA or NDP52 under conditions described in the Methods. Error bars represent SEM.

(d) Velocity histogram from sliding filament assay with MVI immobilised through the stated concentrations of NDP52.

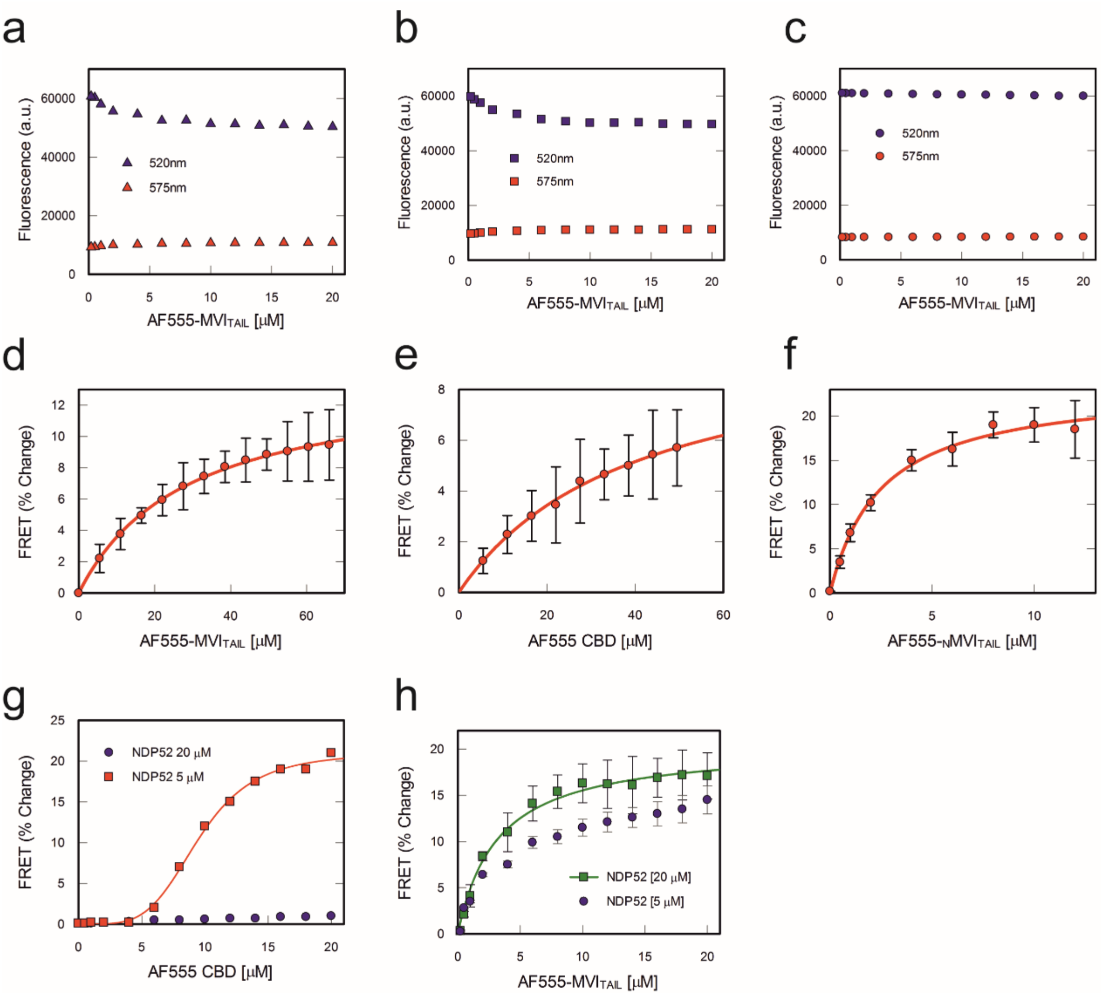

Supplementary Figure 7.

(a) Raw fluorescence intensity data for titration of AF555-MVI$_{TAIL}$ against FITC-MVI$_{TAIL}$ in the presence of 20 µM NDP52. (a-c) Blue is FITC intensity and Red is AF555 intensity.

(b) Raw fluorescence intensity data for titration of AF555-MVI$_{TAIL}$ against FITC-MVI$_{TAIL}$ in the presence of 20 µM DNA/NDP52.

(c) Raw fluorescence intensity data for titration of AF555-MVI$_{TAIL}$ against FITC-MVI$_{TAIL}$.

(d) FRET titration of AF555-MVI$_{TAIL}$ against FITC-MVI$_{TAIL}$ over a larger concentration range.

(e) FRET titration of AF555-CBD against FITC-CBD.

(f) FRET titration of AF555-$_{N}$MVI$_{TAIL}$ against FITC- $_{N}$MVI$_{TAIL}$.

(g) FRET titration of AF555-CBD against FITC-CBD in the presence of 5 µM or 20 µM NDP52. For F-G, fitting was performed as described in the Methods.

(h) FRET titration of AF555-MVI$_{TAIL}$ against FITC-MVI$_{TAIL}$ in the presence of 5 µM or 20 µM NDP52.

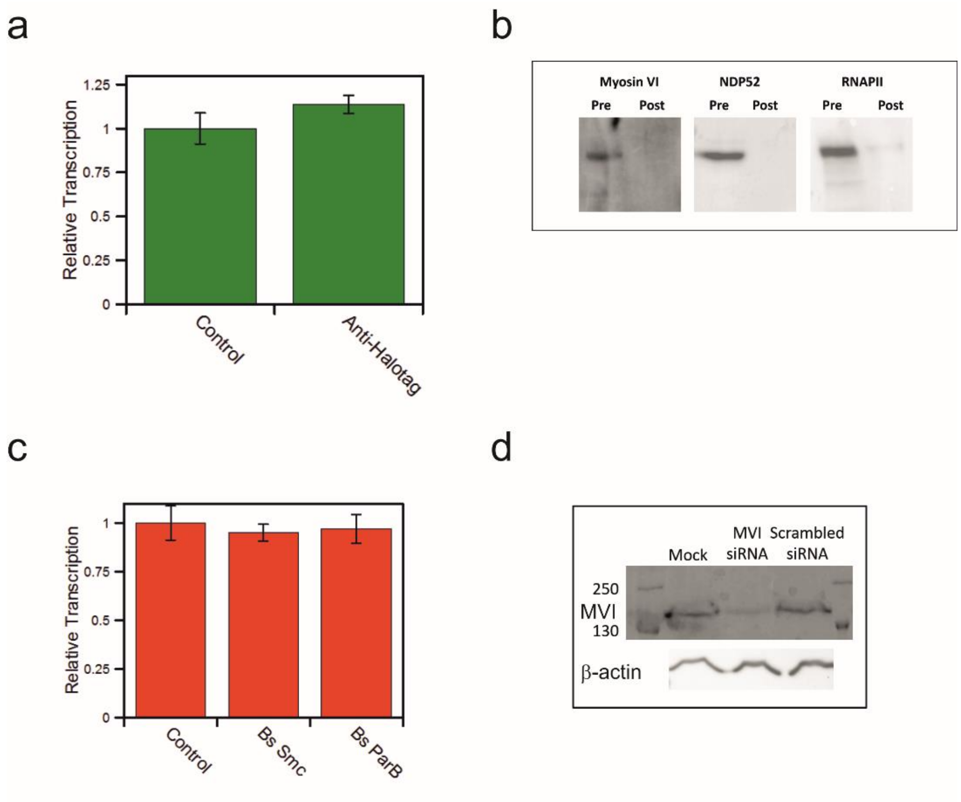

Supplementary Figure 8.

(a) *In vitro* transcription by HelaScribe extracts. A control reaction was performed under standard manufacturer’s instructions. Antibody depletion was performed as described in Methods. Here, Anti-Halotag represents an antibody negative control to highlight the specific isolation of target proteins. Samples were normalized to a non-depleted control reaction (error bars represent SEM).

(b) Confirmation of antibody depletion. Western-blots are shown confirming the presence of MVI, NDP52 and RNAPII in the HelaScribe extracts (Pre). Depletion of the proteins by the corresponding antibodies is shown in the Post samples.

(c) *In vitro* transcription by HelaScribe extracts in the presence of competitor *Bacilus subtilis* Smc and ParB at 25 μM. Both proteins were used as controls for DNA binding which may block transcription. As no decrease in transcription occurred, we conclude that the decrease in transcription observed with the CBD is related to its interaction with the RNAPII complex and not the interference by DNA binding. Samples were normalized to the control sample in Fig. 6e (error bars represent SEM).

(d) siRNA Knock-down of myosin VI. Western-blot on myosin VI in MCF7 cells under mock conditions and following transfection with MVI siRNA and control scrambled siRNA.

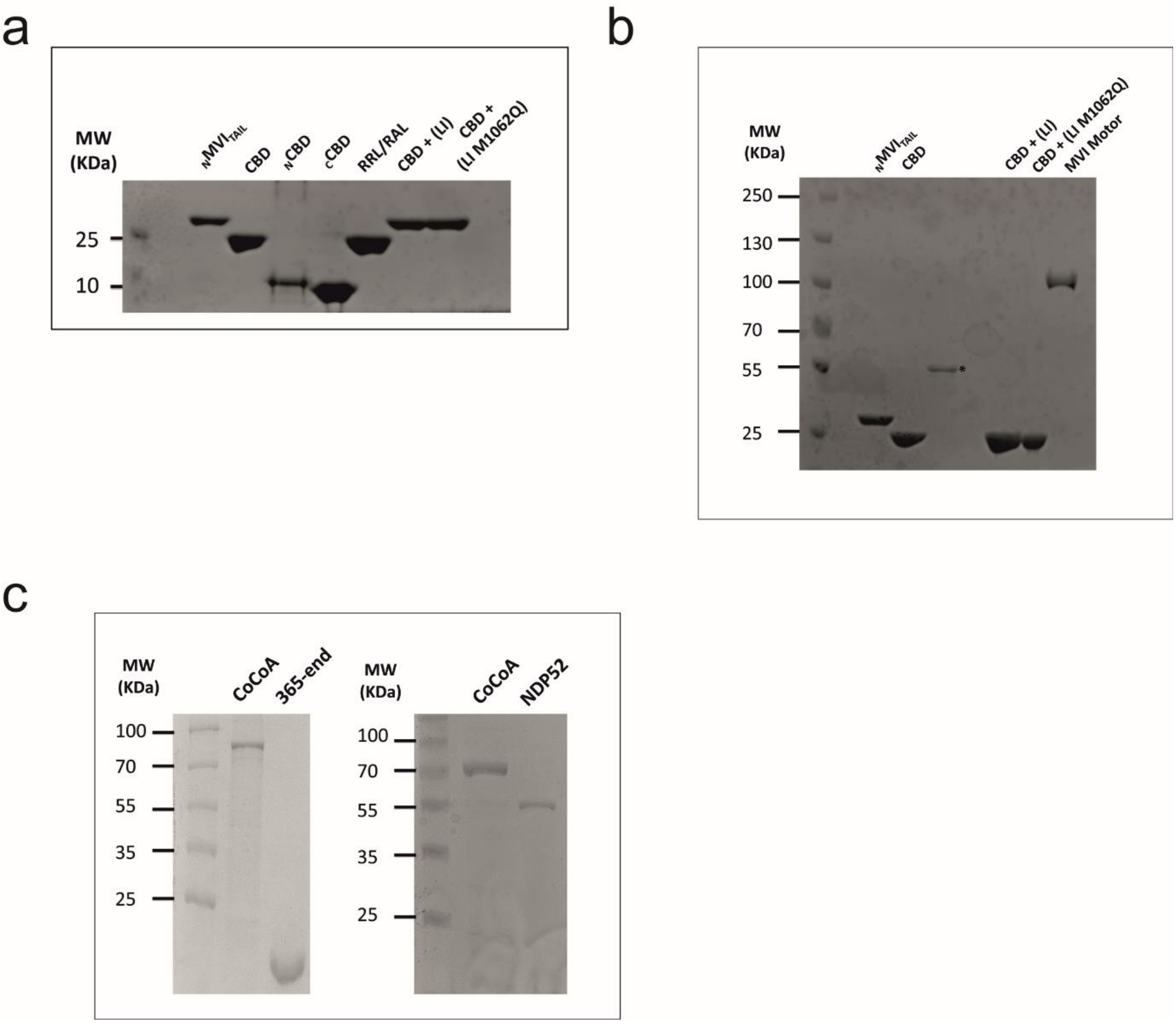

Supplementary Figure 9.

(a-b) Loading controls of recombinant proteins for assays performed in Fig. 4f and 7c.

(c) Loading controls of recombinant proteins for assay performed in Fig. 6c.

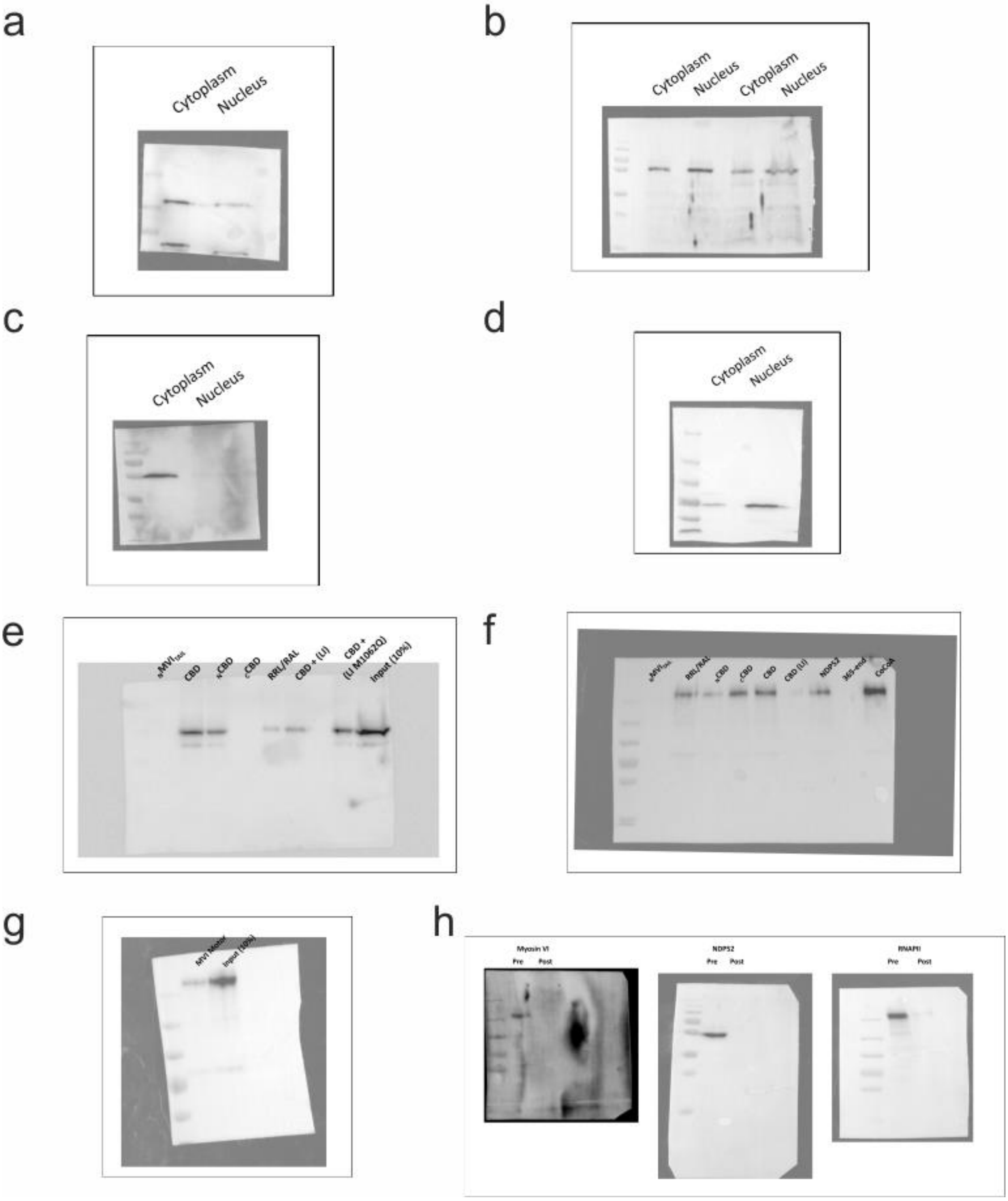

Supplementary Figure 10.

Uncropped Western-blots. (a) MVI From Fig. 1c. (b) NDP52 From Fig. 4c. (c) Tubulin from cell fractionation Fig. 1c and 4c. (d) Lamin B from cell fractionation Fig. 1c and 4c. (e) From Fig. 4f. (f) From Fig. 6c and 7c. (g) From Fig. 7c. (h) From Supplementary Fig. 8b.

Supplementary Table 1. DNA Binding Prediction

Residue	Amino acid	Output	Prediction	Confidence (0-1)
80	R	1.6914	+	0.9539
83	K	1.4826	+	0.9152
85	R	2.0163	+	0.9862
118	K	1.5894	+	0.9373
205	R	1.6289	+	0.9447
276	R	1.7193	+	0.9594
280	R	1.9585	+	0.9825
365	K	1.6639	+	0.9493
368	S	1.4254	+	0.9005
393	R	1.854	+	0.9705
632	K	1.4439	+	0.9023
667	R	1.4925	+	0.9189
708	R	1.9088	+	0.9788
792	R	1.7813	+	0.965
825	R	1.5331	+	0.9309
831	R	1.4709	+	0.9124
832	R	1.3556	+	0.8839
834	K	1.1633	+	0.8166
847	K	1.1978	+	0.8341
849	R	1.2752	+	0.8553
886	K	1.1943	+	0.8313
937	R	1.2846	+	0.8627
940	R	1.1816	+	0.824
948	R	1.1761	+	0.823
1035	R	0.9922	+	0.8235
1036	R	1.038	+	0.8475
1044	K	0.9667	+	0.806
1049	T	0.9875	+	0.8217
1050	K	1.0849	+	0.8714
1055	S	0.9608	+	0.8041
1056	K	1.1173	+	0.8862
1063	R	1.1307	+	0.8917
1085	R	1.0471	+	0.8548
1094	S	1.4206	+	0.8995
1095	K	1.9281	+	0.9816
1096	N	1.3317	+	0.8774
1097	K	1.4436	+	0.9023
1098	K	1.7798	+	0.965
1099	R	1.6503	+	0.9484
1100	N	1.2231	+	0.8433
1106	R	1.0999	+	0.8797
1109	K	1.507	+	0.9235
1112	T	1.0336	+	0.8447
1126	R	1.0418	+	0.8502
1137	R	1.1627	+	0.8147
1155	S	1.1766	+	0.823
1156	K	1.539	+	0.9318
1157	K	1.1108	+	0.8797
1158	K	1.5536	+	0.9336
1205	T	0.9608	+	0.8041
1206	R	1.8287	+	0.9687
1207	K	1.1256	+	0.8908
1208	R	1.4658	+	0.9115
1237	R	1.4731	+	0.9134
1240	R	1.7045	+	0.9548
1242	T	1.1889	+	0.8286
1243	Y	1.2152	+	0.8424
1245	T	1.2904	+	0.8645

Supplementary Table 2. DNA Binding dissociation constants.

Construct	DNA Substrate	Conditions	Figure	*Kd* (μM)
MVI	ds40		2a	3.5 +/- 0.7
Motor1-814	ds40		2a	-
MVITAIL	ds40		2a	2.5 +/- 0.4
NMVITAIL	ds40		2b	1.2 +/- 0.35
CBD	ds40		2b	0.1 +/- 0.02
CBD ΔΣιτεA	ds40		2e	0.11 +/- 0.04
NCBD	ds40		2e	1.3 +/- 0.7
CCBD	ds40		2e	0.12 +/- 0.03
CCBD ΔΣιτεB	ds40		2f	0.7 +/- 0.18
CCBD ΔΣιτεX	ds40		2f	0.5 +/- 0.15
MVITAIL (W1221A)	ds40		3e	0.3 +/- 0.03
CBD	ds15		Sup Fig 2a	0.5 +/- 0.045
CBD	ss20		Sup Fig 2a	0.11 +/- 0.035
CBD	dT20		Sup Fig 2a	0.12 +/- 0.025
CBD	ss40		Sup Fig 2a	0.12 +/- 0.035
MVI	ds40	5 μM F-actin	Sup Fig 3f	2.4 +/- 1.3
MVI	ds40	1 mM Ca2+	Sup Fig 3f	1.7 +/- 0.9
MVI	ds40	2 mM ATP	Sup Fig 3f	2.5 +/- 1.1
MVI	ds40	F-actin + ATP	Sup Fig 3f	2.7 +/- 1.3
NDP52	ds40		Sup Fig 4f	0.08 +/- 0.025

Supplementary Table 3. Myosin VI – DNA Pre-steady-state kinetics.

Construct	Association rate constant (μM^{-1} s^{-1})	Dissociation rate constant (s^{-1})
MVI	9.1 +/- 1.5	21.3 +/- 3.3
MVITAIL	9.4 +/- 1	25.6 +/- 2.2
NMVITAIL	17.4 +/- 2.2	27.8 +/- 4.7
CBD	42.2 +/- 3.8	13.3 +/- 3.9

Supplementary Table 4. Protein-protein dissociation constants.

Construct A	Construct B	Technique	Conditions	Figure	*Kd* (μM)
NMVITAIL	CBD	FRET		3a	4.5 +/- 0.6
NMVITAIL	CBD	Anisotropy		Sup Fig 3d	4 +/- 0.4
CBD	NMVITAIL	Anisotropy		Sup Fig 3d	4.2 +/- 0.4
NDP52	MVITAIL	FRET		4d	3.2 +/- 1.2
NDP52	MVITAIL (LI)	FRET		4d	9.8 +/- 4.3
NDP52	MVITAIL (RRL/ARL)	FRET		4d	4.6 +/- 1.2
NDP52	MVITAIL (RRL/RAL)	FRET		4d	55 +/- 23
NDP52	MVITAIL (RRL/RRA)	FRET		4d	21 +/- 11
NDP52	MVITAIL (RRL/AAA)	FRET		4d	63 +/- 15
AF555-MVITAIL	FITC-MVITAIL	FRET		5a	27 +/- 4.3
AF555-MVITAIL	FITC-MVITAIL	FRET	20 μM NDP52	5a	2.3 +/- 0.8
AF555-MVITAIL	FITC-MVITAIL	FRET	20 μM DNA	5a	29 +/- 5
AF555-MVITAIL	FITC-MVITAIL	FRET	20 μM NDP52/DNA	5a	2.5 +/- 1.2
AF555-CBD	FITC-CBD	FRET		Sup Fig 5g	58 +/- 9.5
AF555-CBD	FITC-CBD	FRET	20 μM NDP52	Sup Fig 5i	51 +/- 7
AF555-NMVITAIL	FITC-NMVITAIL	FRET		Sup Fig 5f	3.1 +/- 1.4

Supplementary Table 5. Recombinant DNA.

**Construct (Residue numbers)**	**Source**
Human pEGFP-C3 myosin VI (large insert) (1-1284)	F. Buss (CIMR)
Human pEGFP-C3 myosin VI M1062Q (large insert) (1-1284)	S. Polo (IFOM)
Human pEGFP-C3 myosin VI (non insert) (1-1253)	F. Buss (CIMR)
Human pEGFP-C3 myosin VI WKSKNKKR/WASANNNR (1-1253)	F. Buss (CIMR)
Human pFastBacHTB GFP NI myosin VI	F. Buss (CIMR)
Xenopus pFastBac1 Calmodulin (1-end)	J. Sellers (NIH)
Bacillus subtilis pET28 Smc hinge	S.Gruber (MPIB)
Bacillus subtilis pET28 ParB (1-end)	S.Gruber (MPIB)
pSG1365	S.Gruber (MPIB)
Human pET151 GST ERalpha (1-end)	Synthetic Gene – This Study
Human pET151 NDP52 (1-end)	Synthetic Gene – This Study
Human pET151 NDP52 (365-end)	Synthetic Gene – This Study
Human pET151 EGFP-MVI_{TAIL}-RFP (814-1253)	Synthetic Gene – This Study
Human pET151 MVI_{TAIL} (814-1253) W1221A	Synthetic Gene – This Study
Human pET151 CBD (1060-end) W1221A	Synthetic Gene – This Study
Human pET151 CoCoA (1-end)	Synthetic Gene – This Study
Human pMX MVI (1184-1253)	Synthetic Gene – This Study
Human pET151 MVI_{TAIL} 814-1253	This Study
Human pET151 $_{N}MVI_{TAIL}$ 814-1060	This Study
Human pET28 CBD 1060-1253	This Study
Human pET28 $_{N}CBD_{1060-1120}$	This Study
Human pET28 $_{C}CBD_{1121-1253}$	This Study
Human pET28 MVI_{TAIL} (large insert) (814-1284)	This Study
Human pET28 CBD (large insert) (1037-1284)	This Study
Human pET28 CBD M1062Q (large insert) (1037-1284)	This Study
Human pFastbacHTB NI MVI (1-1253)	This Study
Human pFastbacHTB $Motor_{1-814}$	This Study
Human pFastbacHTB $Motor_{1-1060}$	This Study
Human pET28 CBD ΔSite A WKSKNKKR/WASANNNR (1060-	This Study
Xenopus pET28 Calmodulin	This Study
Human pET28 $_{C}$CBD ΔSite B SKKK/AAAA	This Study
Human pET28 $_{C}$CBD ΔSite C TRKR/AAAA	This Study
Human pET28 CBD RRL/RAL	This Study
Human pET28 CBD LQSLL/AQSAA	This Study
Human pET28 MVI_{TAIL} RRL/RAL	This Study
Human pET28 MVI_{TAIL} RRL/ARL	This Study
Human pET28 MVI_{TAIL} RRL/RRA	This Study
Human pET28 MVI_{TAIL} RRL/AAA	This Study

Supplementary Table 6. Primers for qPCR.

Sequence	Use
CATGGAGAACAAGGTGATCTG	TFF1/PS2 qPCR For
CACTGTACACGTCTCTGTCTG	TFF1/PS2 qPCR Rev
ATGGGAAATTCTTACGCTGGAC	GREB1 qPCR For
CACTCGGCTACCACCTTCT	GREB1 qPCR Rev
AGAGCTACGAGCTGCCTGAC	Human B-Actin qPCR For
AGCACTGTGTTGGCGTACAG	Human B-Actin qPCR Rev
AAGCTTCGATGATGGGCTTA	ESR1 qPCR For
AGGTGGACCTGATCATGGAG	ESR1 qPCR Rev
CCGAGCTCATCAGTGATGAGGC	Myosin VI qPCR For
CCAAGCATGATACACTTTTAGTCTCC	Myosin VI qPCR Rev
AAGGGCATCGACTTCAAGGA	CMV GFP RT-qPCR For In vitro Transcription
GGCGGATCTTGAAGTTCACC	CMV GFP RT-qPCR Rev In vitro Transcription

Supplementary Table 7. DNA Substrates.

Sequence	Use
TTAGTTGTTCGTAGTGCTCGTCTGGCTCTGGATTACCCGC*	ds40 A 3'FAM
GCGGGTAATCCAGAGCCAGACGAGCACTACGAACAACTAA	ds40 B
TTAGTTGTTCGTAGTGCTCGTCTGGCTCTGGATTACCCGC*	ss40 A 3'FAM
TCTGGCTCTGGATTACCCGC*	ds20 A 3'FAM
GCGGGTAATCCAGAGCCAGA	ds20 B
TCTGGCTCTGGATTACCCGC*	ss20 A 3'FAM
TTAGTTGTTCTCTGG*	15 bp A 3'FAM
CCAGAGAACAACTAA	15 bp B
TTTTTTTTTTTTTTTTTTTT	dT_{20}

Supplementary Table 8. PCR Primers.

Sequence	Use
AGCTAGCTGCATGGCTGACCAACTG	pET28 Calmodulin For
TTTTGCGGCCGCTCACTTTGCTGTCATC	pET28 Calmodulin Rev
CTAGGCGGCCGCCCGAGGATGGAAAGCCCGTTTG	pFastbacHTB NI Myosin VI
TTTTCTCGAGTTATTTCAACAGGTTCTGCAGCATG	pFastbacHTB NI Myosin VI
CACC GAAGCCTGCATTAAAATGC	MVI_{TAIL} 814-1253 For
CTATTTCAACAGGTTCTGCAGCAT	MVI_{TAIL} 814-1253 Rev
CTAGGCGGCCGCCCGAGGATGGAAAGCCCGTTTG	pFastBacHTB NI MVI
TTTTCTCGAGCTAGCATTTTAATGCAGGCTTC	pFastBacHTB NI MVI
CTAGGCGGCCGCCCGAGGATGGAAAGCCCGTTTG	pFastBacHTB NI MVI
TTTTCTCGAGCTAGATGGTATCACGTAGTTCTGC	pFastBacHTB NI MVI
AGCTAGCGCAGAACTACGTGATACCATC	CBD For
TTTTGCGGCCGCTATTTCAACAGGTTCTGCAGCAT	CBD Rev
AGCTAGCGCAGAACTACGTGATACCATC	$_{N}$CBD For
TTTTGCGGCCGCTATGCTGGGTTTTGCTGAGG	$_{N}$CBD Rev
AGCTAGCGCTCAGATTCCTGCCAGG	$_{C}$CBD For
TTTTGCGGCCGCTATTTCAACAGGTTCTGCAGCAT	$_{C}$CBD Rev
AGCTAGCGGGGCAGAACTCAGCACTG	CBD Plus LI For
TTTTGCGGCCGCTATTTCAACAGGTTCTGCAGCAT	CBD Plus LI Rev
CACC GAAGCCTGCATTAAAATGC	$_{N}MVI_{TAIL}$ For 814-1060
CTAGATGGTATCACGTAGTTCTGC	$_{N}MVI_{TAIL}$ Rev 814-1060
AGCTAGCGCCGAACTGCGTGATACCA	CBD W1221A For
TTTTGCGGCCGCTATTATTTCAGCAGGCTCTGCAGC	CBD W1221A Rev
CTTGCAGAGAAGAATTTCATGCGGCAGCAAAAGTGTATCATGC	RRL/AAA For
GATTTCCAAGCATGATACACTTTTGCTGCCGCATGAAATTCTTC	RRL/AAA Rev
CTTGCAGAGAAGAATTTCATAGGAGAGCAAAAGTGTATCATGC	RRL/RRA For (L1118A)
GATTTCCAAGCATGATACACTTTTGCTCTCCTATGAAATTCTTC	RRL/RRA Rev (L1118A)
CTTGCAGAGAAGAATTTCATGCGAGACTAAAAGTGTATCATGC	RRL/ARL For (R1116A)
GATTTCCAAGCATGATACACTTTTAGTCTCGCATGAAATTCTTC	RRL/ARL Rev (R1116A)
GCAGAGAAGAATTTCATAGGGCACTAAAAGTGTATCATGCTTG	RRL/RAL Rev (R1117A)
CCAAGCATGATACACTTTTAGTGCCCTATGAAATTCTTCTCTGC	RRL/RAL Rev (R1117A)
GACCCTCAGAGTGCGGCAGCAGGCTGGTGGTATGC	SKKK/SAAA For
GCATACCACCAGCCTGCTGCCGCACTCTGAGGGTC	SKKK/SAAA Rev
GACTGGCCTGACTCGGAAGCGTGGTGCTGAGATCTTG	TRKR/TAAA For
CAAGATCTCAGCACCACGCTTCCGAGTCAGGCCAGTC	TRKR/TAAA Rev
GCCATGGCGCAGAACGCGGCGAAATAAGCCGAAG	LQSLL/AQSAA For
CTTCGGCTTATTTCGCCGCGTTCTGCGCCATGGC	LQSLL/AQSAA Rev
GATCGATAGTACATAAGGATTTCCTTACGCG	500bp 5'Bio-Teg
CCAATTTTCGTTTGTTGAACTAATGGGTGC	500 bp Rev
