# Supplementary material for: NDP52 activates nuclear myosin VI to enhance RNA polymerase II transcription
Source: Nat Commun. 2017 Nov 30;8:1871. doi: 10.1038/s41467-017-02050-w (PMC5707354; doi:10.1038/s41467-017-02050-w)
Supplement: Supplementary file 2 — Description of Additional Supplementary Files [file 41467_2017_2050_MOESM2_ESM.pdf]

Description of Additional Supplementary Files

File Name: Supplementary Movie 1
Description: Immunofluorescence staining against MVI in HeLa cell cropped to the nucleus, as shown in Fig. 1b. Scale bar is 1 μm.

File Name: Supplementary Movie 2
Description: Immunofluorescence staining against MVI in isolated HeLa cell nuclei, as shown in Fig. 1b. Scale bar is 1 μm.

File Name: Supplementary Movie 3
Description: Nuclei stained with the DNA dye Hoechst 33342 and the lipophilic dye DID, as shown in Supplementary Fig. 1. Scale bar is 1 μm.

File Name: Supplementary Movie 4
Description: Second example of nuclei stained with the DNA dye Hoechst 33342 and the lipophilic dye DID, as shown in Supplementary Fig. 1. Scale bar is 1 μm.

File Name: Supplementary Movie 5
Description: 3D projection movie of the nucleus presented in Video 4.

File Name: Supplementary Movie 6
Description: Fixed HeLa cells stained with Rhodamine-Phalloidin and Hoechst 33342. The image is cropped to the nucleus, as shown in Supplementary Fig. 2a. Scale bar is 1 μm.

File Name: Supplementary Movie 7
Description: Immunofluorescence staining against NDP52 in HeLa cell cropped to the nucleus, as shown in Fig. 4b. Scale bar is 1 μm.

File Name: Supplementary Movie 8
Description: Immunofluorescence staining against NDP52 in isolated HeLa cell nuclei, as shown in Fig. 4b. Scale bar is 1 μm.
